# Genome of the tropical plant *Marchantia inflexa*: implications for sex chromosome evolution and dehydration tolerance

**DOI:** 10.1038/s41598-019-45039-9

**Published:** 2019-06-19

**Authors:** Rose A. Marks, Jeramiah J. Smith, Quentin Cronk, Christopher J. Grassa, D. Nicholas McLetchie

**Affiliations:** 1 Department of Biology, University of Kentucky, 101 Thomas Hunt Morgan Building, Lexington, KY 40506 USA; 2 Department of Botany, University of British Columbia, 6270 University Boulevard, Vancouver, BC V6T 1Z4 Canada; 3 Present Address: Department of Organismic and Evolutionary Biology, Harvard University Herbaria, 22 Divinity Avenue, Cambridge, MA 02138 USA

**Keywords:** Genomics, Plant evolution

## Abstract

We present a draft genome assembly for the tropical liverwort, *Marchantia inflexa*, which adds to a growing body of genomic resources for bryophytes and provides an important perspective on the evolution and diversification of land plants. We specifically address questions related to sex chromosome evolution, sexual dimorphisms, and the genomic underpinnings of dehydration tolerance. This assembly leveraged the recently published genome of related liverwort, *M*. *polymorpha*, to improve scaffolding and annotation, aid in the identification of sex-linked sequences, and quantify patterns of sequence differentiation within *Marchantia*. We find that genes on sex chromosomes are under greater diversifying selection than autosomal and organellar genes. Interestingly, this is driven primarily by divergence of male-specific genes, while divergence of other sex-linked genes is similar to autosomal genes. Through analysis of sex-specific read coverage, we identify and validate genetic sex markers for *M*. *inflexa*, which will enable diagnosis of sex for non-reproductive individuals. To investigate dehydration tolerance, we capitalized on a difference between genetic lines, which allowed us to identify multiple dehydration associated genes two of which were sex-linked, suggesting that dehydration tolerance may be impacted by sex-specific genes.

## Introduction

Bryophytes (mosses, liverworts, and hornworts) are living representatives of an early diverging land plant lineage^1,2^ and they provide an important landmark for comparative phylogenetics. Although the exact relationships among bryophyte lineages are currently contested, the earliest fossil evidence assigned to a bryophyte is liverwort-like with dorsiventral complex thallus morphology and a leafless gametophyte^3–5^, suggesting that liverworts retain a large suite of ancestral characters not conserved in other land plants. Importantly, building a fundamental understanding of genomic patterns can be readily accomplished by working with bryophytes because of their small genomes^6^, many of which contain comparatively few paralogous duplications of regulatory genes^2^. Thus far, genome assemblies have been developed for a number of bryophyte species (including the mosses *Physcomitrella patens*^7^, and *Sphagnum fallax*^8^, the liverwort *Marchantia polymorpha*^2^, and the hornwort *Anthoceros agrestis*^9^), and more are underway (*Takakia lepidozioides*, *Ceratodon purpureus*, *Funaria hygrometrica*^10^ and *Syntricia caninervis*). Relatively few assemblies of bryophyte mitochondria^11–15^ and chloroplasts^16–18^ are available compared to other plant lineages, yet mitochondrial genomes of bryophytes tend to be less complex than those of tracheophytes, having no large repeated sequences and limited recombination^19^. Genome sequencing efforts of additional bryophyte taxa will provide critical insight into more recent evolutionary changes within these lineages and may help to better resolve ancestral states.

In this context, we targeted the tropical liverwort, *Marchantia inflexa* (Nees & Mont) for sequencing and assembly. *Marchantia inflexa* is a New World liverwort that is distributed throughout Central America and the Caribbean, from northern Venezuela to the southern United States^20^. *Marchantia inflexa is* 68–126 million years diverged from the well-studied sister species *M*. *polymorpha*^21^. *Marchantia inflexa* is dioecious (has unisexual individuals), has eight autosomes and one female (U) or male (V) sex chromosome, and reproduces sexually by spores, or asexually by fragmentation or the formation of gemmae (specialized asexual propagules). *Marchantia inflexa* typically grows on rock and soil surfaces along stream banks in tropical forests, but can also colonize more exposed and disturbed sites along roads. Vegetative growth produces a dichotomously branching thallus mat with dorsiventral organization, and the haploid gametophyte is the dominant life phase. *Marchantia inflexa* is a useful model to investigate sexual dimorphisms, population sex ratios, and stress tolerance because prior work has established that *M*. *inflexa* exhibits a considerable degree of sexual dimorphism^22,23^, variable population sex ratios^22,24,25^, and fluctuating stress tolerance^26,27^.

Bryophytes harbor a high proportion of dioecious species. Nearly half of all extant mosses, and approximately two-thirds of liverworts are dioecious^28^. In many bryophytes (including *M*. *inflexa*) the reported sex ratio is often female biased^24,25,29^, and in *M*. *inflexa*, this may be related to females’ superior ability to recover from drying events^26,27^, faster growth rate^23^, or the increased establishment of female gemmae^27^. However, true population sex ratios are largely unknown, except for the few cases where genetic sex markers have been developed and utilized^30–32^. Typical methods for assessing sex ratios depend on counting the number males and females with visible sex organs and using this to infer the underlying population sex ratio. However, this approach fails to account for plants not currently displaying sex organs and assumes that the sex ratio of vegetative plants is equivalent to that of plants with sex organs^33^. This assumption may not hold true in natural settings. In fact, for both *M*. *polymorpha* and *M*. *inflexa* (where sex organ development can be artificially induced) the timing of reproductive development is sex-specific^34^ and some individuals never produce sex organs (unpublished data).

The reproductive biology of bryophytes (with the haploid gametophyte being the dominant life stage) provides a unique perspective on the evolution of sex-linked genes, as the female (U) and male (V) sex chromosomes are present at the same copy number (1N) as autosomal chromosomes for the majority of the organism’s life cycle and are subject to haploid selection. Sex chromosome evolution in diploid dominant systems has received considerable research attention. However, less is known about the forces shaping sex chromosomes in haploid dominant systems^35^, and the ramifications of haploid selection on sex chromosomes may have unique consequences. For example, exposure to haploid selection should reduce the prevalence of deleterious mutations^36,37^ and could allow beneficial mutations fix more rapidly^36^. However, lack of recombination on UV sex chromosomes could lead to degeneration on UV chromosomes^35,36^, as has been observed in XY and ZW chromosomes. Further, the smaller effective population size of sex chromosomes relative to autosomes may increase the impact of genetic drift, further influencing adaptive evolution of sex-specific genes^38^. The extent to which these forces shape sex chromosome evolution in haploid dominant systems is not well understood, but the numerous dioecious bryophyte taxa provide novel opportunities to test related questions.

Stresses caused by environmental fluctuations are accentuated in plants due to their sessile nature. Consequently, numerous tolerance mechanisms have evolved to combat environmental pressures, many of which have potential translational utility. Some of these stress tolerance traits, such as embryo retention (allowing for the development and dispersal of desiccation tolerant spores), UV radiation, desiccation, heat, and freezing tolerance^2,39^ may have facilitated the transition from aquatic to terrestrial environments by early plants. Many extant bryophytes retain these early stress tolerance mechanisms, allowing them to occupy marginal niches (characterized by nutrient poor substrates^40^, toxic concentrations of metals^41^, variable light^42^ and moisture levels^43^). Consequently, bryophytes are particularly informative with respect to understanding the evolutionary history and physiological strategies of stress tolerance.

Desiccation tolerance (DT)^43^ in particular, has important translational utility. A number of studies have described the genomes^44–47^ and transcriptomes^48–55^ of DT plants, and the amassing data provide a strong foundation on which to construct our understanding of DT. These studies have demonstrated that DT is a complex multigenic trait^44,48,51,56,57^, and that there are multiple means of achieving DT^58^. The genetic basis of DT, although not entirely described, may derive from regulatory differences in gene expression pathways^45^, increased copy number of anahydrobiosis related genes^59^, or differences in the structural organization of these genes^44^. However, more studies are needed to resolve the specifics of DT mechanisms, and should include work on species spanning a wide phylogenetic range and degree of tolerance levels (such as the intermediate trait of dehydration tolerance (DhT also dehydration tolerant)^26,60^). *Marchantia inflexa* is DhT, which provides an important opportunity to enhance our understanding of the evolution of this intermediate trait.

Growing genomic resources for bryophytes provide novel opportunities to conduct comparative studies within these lineages, which are particularly well suited to addressing questions related to sex chromosome evolution, sex differences, and stress tolerance adaptations. Here, we aimed to characterize patterns of sequence divergence between the thalloid liverworts *M*. *inflexa* and *M*. *polymorpha*, define genetic sex markers, and investigate the genomic basis of intraspecific variation in DhT. Our analyses indicate that the greatest sequence conservation between *M*. *inflexa* and *M*. *polymorpha* is among chloroplast genes, likely due to the conservation of plastid function across lineages. Conversely, we show that mitochondrial genes are highly divergent, which may be related to reduced recombination of mitochondrial genomes (as observed in *M*. *polymorpha*)^19^, or variable mutation rates^61^. Sex-linked genes exhibit signatures of strong diversifying selection, relative to autosomal genes. Interestingly, this is driven primarily by male-specific (V) genes, which we speculate is related to strong selection on male traits to maintain species recognition. Because sperm is broadcast indiscriminately and water dispersed in *Marchantia*, pressure to maintain species recognition is expected to be particularly strong on genes related to sperm characteristics^62^. Although females could be subject similar selective pressures, our analyses indicate that selection is acting primarily on male-specific genes in *M*. *inflexa*. Putatively sex-specific sequences were identified and leveraged to develop diagnostic markers for genetic sex in *M*. *inflexa*. Regarding DhT, we detect higher copy number of dehydration associated genes in the tolerant female genotype compared to the less tolerant male genotype. Interestingly, some of these DhT genes are putatively sex-linked, offering a possible explanation for elevated DhT in *M*. *inflexa* females^26,27^. The remaining DhT genes appear to be located on autosomes and are expressed at similar levels in both sexes, which may contribute to the changing patterns of DhT that have been observed^63^.

## Results

### Genome assembly and annotation

Whole-genome sequencing of *M*. *inflexa*, yielded 127,147,280 male reads and 133,660,960 female reads (after quality filtering). The combined male and female k-mer distribution indicated a coverage of ~24x, but showed a large quantity of unique and low abundance k-mers, suggestive of contaminating organisms (see Supplementary Fig. S1). In our efforts to characterize the source of these low abundance k-mers, we detected a diverse community of microbes, consistent with recent descriptions of *M*. *inflexa* microbial associations^29^. After removal of putative microbial sequences, we assembled the remaining sequence reads to generate the draft assembly M_inflexa_v1.1. The resulting scaffolds were assigned to super-scaffolds by alignment with the *M*. *polymorpha* reference genome, allowing us to coalesce the assembly into 300 super-scaffolds. In total 7,747 *M*. *inflexa* scaffolds covering a total length of 81,634,927 bp were successfully mapped to the *M*. *polymorpha* genome. Unmapped *M*. *inflexa* scaffolds were appended to the supper-scaffold assembly. The resulting assembly consists of 41,556 scaffolds, covering a total of 208,839,958 bp, with and N50 of 11,144 bp and a longest scaffold length of 2,829,880 bp. This Whole Genome Shotgun project (M_inflexa_v1.1) has been deposited at DDBJ/ENA/GenBank under the accession QLSQ00000000. The version described in this paper is version QLSQ01000000.

Assessment of assembly completeness (performed with BUSCO^64^) indicated that 54.4% (783) of the 1,440 presumptively universal single-copy orthologs from the plant set of OrthoDB v9 were present in the *M*. *inflexa* genome assembly. Another 3.5% (51) orthologs were present, but fragmented. In comparison, a parallel assessment of the *M*. *polymorpha* v3.1 assembly, found that 60.2% (867) of these same genes were complete, and 2.9% (42) were fragmented in *M*. *polymorpha*. Both of these estimates are rather low, suggesting that there may be inherent limitations associated with BUSCO as has been observed for other deeply diverged lineages^65,66^. Still, we find these assessments to be informative in a comparative context within *Marchantia*.

**Figure 1:**
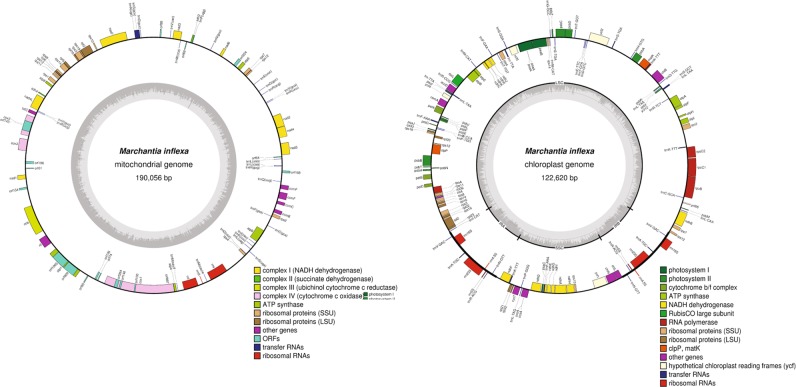
Assembled mitochondrial and chloroplast sequences of *Marchantia inflexa*. The inner circle depicts nucleotide content (G/C in dark gray and A/T in light gray). Inverted repeats (IRA and IRB) along with large (LSC) and small (SSC) single copy regions are indicated on the chloroplast inner circle. The outer circle shows annotated genes that are color coded by function. Genes located on the outside of the circle are transcribed in a clockwise direction, and those on the inside of the circle are transcribed counterclockwise.

Assembly of *M*. *inflexa* plastids generated nearly complete mitochondrial and chloroplast sequences (Fig. 1). The mitochondria of *M*. *inflexa* is 190,056 bp and the chloroplast is 122,620 bp. The complete mitochondrial and chloroplast sequences are available at FigShare (10.6084/m9.figshare.6639209.v1).

### Annotation

Gene annotation of the *M*. *inflexa* draft genome utilized *de novo* gene finding in combination with the lift-over of all *M*. *polymorpha* annotations for orthologous genes. Lift-over annotations from *M*. *polymorpha* to *M*. *inflexa* resulted in the annotation of 10,005 orthologous proteins within the *M*. *inflexa* assembly. *De novo* gene finding efforts identified 13,546 predicted proteins, 9,194 of which had identifiable orthologs across *M*. *polymorpha*, *P*. *patens*, *A*. *thaliana*, and refseq. After removal of all redundant annotations in the *de novo* and lift-over annotations the combined set of annotations consists of 11,687 predicted proteins. Not surprisingly, the highest homology was observed between *M*. *inflexa* and *M*. *polymorpha* with substantially less homology between *M*. *inflexa*, *P*. *patens* and *A*. *thaliana*, reflecting the estimated divergence times among these species (divergence time between *M*. *inflexa* and *M*. *polymorpha* is 68–126 MYA; for *M*. *inflexa* and *P*. *patens* it is 425–557 MYA; and for *M*. *inflexa* and *A*. *thaliana* it is 481–584 MYA^21^).

### Sequence similarity between *M*. *inflexa* and *M*. *polymorpha*

**Figure 2:**
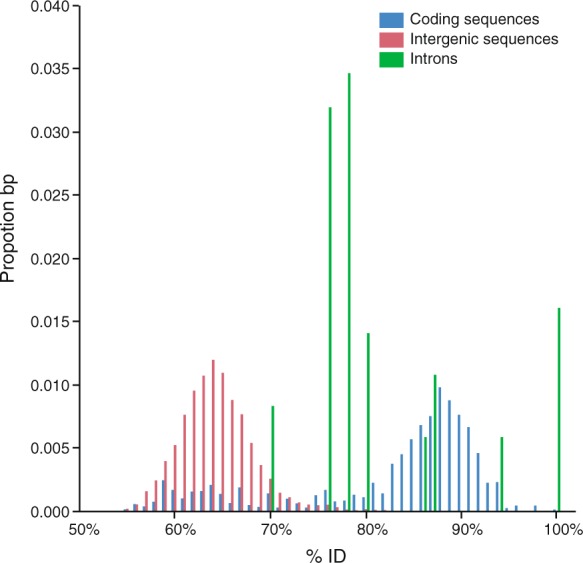
The similarity between *Marchantia inflexa* and *M*. *polymorpha* coding sequences (CDS), introns, and intergenic sequences is shown as proportion base pairs (bp) at a % identity (%ID) ranging from 50–100%. CDS have higher overall similarity compared to introns and intergenic sequences. There was a significant effect of sequence type on %ID. Total bp of each sequence type is: CDS = 629,624 bp; introns = 1,087 bp; and intergenic = 21, 983, 100 bp.

To investigate genome evolution within *Marchantia* we measured sequence divergence between *M*. *inflexa* and *M*. *polymorpha*. Initially, we compared nucleotide differentiation among coding sequences (CDS), introns, and intergenic regions to estimate general patterns of divergence between lineages (Fig. 2). Comparison of orthologous CDS, introns, and intergenic sequences, revealed that (not surprisingly) intergenic sequences were the least conserved (64.5% ± 0.009%), introns were intermediate (81.8% ± 0.008%), and CDS were the most conserved (82.4% ± 0.001%) (Fig. 2). There was a significant effect of sequence type on %ID (F_2,40000_ = 39756, p < 0.0001). Patterns of sequence divergence between *M*. *inflexa* and *M*. *polymorpha* fit general expectations that CDS should exhibit higher sequence similarity compared to introns and intergenic sequences. That being said, we observed surprisingly high sequence conservation among some introns, which we speculate is related to the relatively short length of *M*. *inflexa* introns, in which functional elements (such as splice sites) may be preferentially retained.

**Figure 3:**
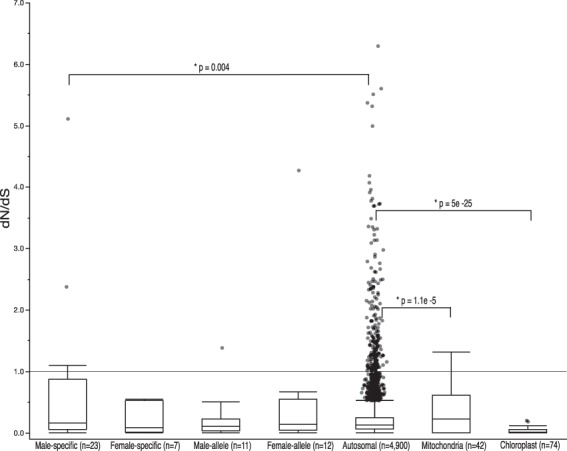
Boxplot of dN/dS values of orthologous coding sequences in *Marchantia inflexa* and *M*. *polymorpha*. There were significant differences among gene types in mean dN/dS. Secondary comparisons reveal significant differences in mean dN/dS among male-specific and autosomal genes, chloroplast and autosomal genes, and mitochondrial and autosomal genes. Statistical tests were performed on log transformed data to satisfy the assumptions of normality, but untransformed numbers are presented here. All dN/dS values > 1 exhibit the signature of diversifying selection.

In order to assess variation in substitution rates across coding sequences, we computed the ratio of non-synonymous to synonymous mutations (dN/dS) for all orthologous CDS of *M*. *inflexa* and *M*. *polymorpha*. The resulting dN/dS values were log transformed to improve normality for statistical testing. Initially, we tested for evidence of contrasting selective pressures among autosomal, sex-linked, and organellar genes. Notably, sex linked genes and autosomes are present at 1 N, whereas copy number of the chloroplast and mitochondria is variable. We computed mean dN/dS and standard error for autosomal genes (0.24 ± 0.01 (n = 4,900)), for sex-linked genes (0.48 ± 0.13 (n = 53)), and for organellar genes (0.14 ± 0.03 (n = 116)). We detected significant differences among groups (F_2,4862_ = 18.54, p < 0.001). Targeted contrasts revealed significant differences among sex-linked and autosomal genes (t_1_ = −2.38, p = 0.018) and among organellar and autosomal genes (t_1_ = 5.58, p = 2.6*e*^−8^). Subsequently, we tested for differences among more specific gene types; subdividing sex-linked genes into male-specific, female-specific, and male and female alleles of genes with both U and V copies. Organellar genes were subdivided into mitochondrial and chloroplast genes. Mean dN/dS of male-specific genes was 0.63 ± 0.23 (n = 23), of female-specific was 0.20 ± 0.09 (n = 7), of male alleles was 0.24 ± 0.12 (n = 11), and of female alleles was 0.56 ± 0.34 (n = 12). Mean dN/dS of chloroplast genes was 0.03 ± 0.01 (n = 74) and of mitochondria was 0.34 ± 0.06 (n = 42). There was an overall effect of gene type on dN/dS (F_2,4862_ = 20.10, p < 0.001). Targeted contrasts revealed significant differences between autosomal genes and male-specific genes (t_1_ = −2.88, p = 0.004), chloroplast genes (t_1_ = 10.39, p = 5*e*^−25^), and mitochondrial genes (t_1_ = −4.40, p = 1.1*e*^−5^) (Fig. 3).

These analyses reveal several genes and pathways that may be under diversifying selection (dN/dS > 1) in *M*. *inflexa* and *M*. *polymorpha*. Sex-linked genes with dN/dS > 1 included the female allele of CCR4-NOT transcription related complex protein (Mapoly0018s0021.1), the male bHLH-MYC2 transcription factor in the involved the jasmonate signaling pathway^67^ (MapolyY_B0018.1), a male-specific phosphatidylinositol-4,5-bisphosphate 3-kinase (MapolyY_A0049.1), two male-specific genes of unknown function (MapolyY_B0032.1 and MapolyY_B0003.1). No chloroplast genes in our analyses had dN/dS > 1, but three mitochondrial open reading frames (orf 84, orf 69, rpl 10) had dN/dS > 1. Of the 243 autosomal genes with dN/dS > 1, 51 had identifiable homologs in the Uniprot database. GO analyses of these genes revealed that many were associated with the cellular components *intracellular*, *cytoplasm*, and *membrane*, the molecular function *catalytic activity* (followed closely by *hydrolase activity* and *transferase activity)*, and the biological processes of *metabolic process* and *cellular process*. A complete list of genes with dN/dS > 1 and associated protein names can be found as Supplementary Table S1.

### Sex marker identification

**Figure 4:**
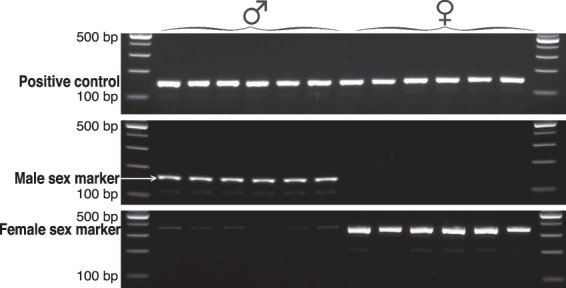
Electrophoresis pattern of sex markers and positive control (actin) amplified in six male and six female plants. Images shown here are sections cropped from two separate gels (see Supplementary Fig. S5 for full length gel images). Male and female markers are presumptive U- and V-linked sequences, respectively. Plants were originally collected from five natural populations spanning a range of environmental conditions. In total, sex markers were validated in nine male and nine female plants, but only six individuals of each sex are shown here.

We identified 4,468 regions (covering 2,234,000 bp) in the *M*. *inflexa* genome assembly with substantial differences in copy number among genetic lines through coverage analysis with DifCover (https://github.com/timnat/DifCover)^65^ (see Supplementary Fig. S2). Of these, 89 were found on scaffolds also containing a predicted protein, 31 of which could be assigned to an identifiable homolog across *M*. *polymorpha*, *P*. *patens*, *A*. *thaliana*, and refseq databases. From this set, we identified five putatively male- and three female-specific sequences that were also orthologous to sequences on the U and V chromosomes in *M*. *polymorpha*. These candidate sex markers were analyzed by PCR in nine males and nine females to verify their fidelity, leading to the validation of one positive marker for each sex (Fig. 4). Other candidate sex markers exhibited non-specific amplification and were therefore discarded. Plants used for validation were originally collected from five distinct populations, suggesting that the markers are robust to genotypic variation. Primer sequences of the validated male and female sex markers are listed in Table 1.

**Table 1:** Primer sequences for validated male and female genetic sex markers for *Marchantia inflexa*.

Marker	Left primer sequence	Right primer sequence
Male marker-98683	CGTTTGATTCGTCTTCTCCAAA	AGCTCTCGTCAGAATAGTCAGG
Female marker-42793	GTCCAGTCTGTGAAGCCGTA	CCTTCTCGTAGACCAGTGCT

### Dehydration tolerance

To address specific hypotheses on DhT we probed *M*. *inflexa* and *M*. *polymorpha* annotated proteins for orthologs to a list of 195 DT genes (compiled from publicly available mRNA sequences of genes expressed under water stress in model DT plants). See Supplementary Table S2 for the accession numbers, species, and studies from which genes were compiled. Of this set of DT genes, 112 had identifiable homologs in *M*. *inflexa* and 141 had identifiable homologs in *M*. *polymorpha*. Our analyses of dN/dS captured 38 of these DT orthologs, one of which (a putative aldehyde dehydrogenase (Mapoly0030s0099.1)) had a dN/dS value > 1. The function of diversification in this gene is unclear, given the lack of evidence for any difference in DhT between these two *Marchantia* species.

Prior studies showed that the male and female *M*. *inflexa* genotypes used for genome assembly have reproducible differences in DhT^26^. Consequently, we aimed to identify DT genes with substantial coverage differences among these two genotypes, presuming that they may impact relative differences in DhT. Of the 112 DT genes detected in *M*. *inflexa*, most had standardized coverage ratios of ~1. However, six genes had considerably higher coverage (log_2_ fold change > |4|) in the highly tolerant female and one had higher coverage in the less tolerant male (Table 2). Specific genes with higher coverage in the tolerant female genotype include a calcium dependent protein kinase (CDPK), glucose related protein 94 (GRP94), the aldehyde dehydrogenase (ALDH) (also identified in dN/dS analyses above), heat shock proteins 70 and 101 (HSP101, HSP70), and superoxide dismutase (SOD). The sole DT gene with higher coverage in the less tolerant male genotype is a heat shock factor 1 (HSF 1). Of the DT genes with coverage difference among genotypes, one (CDPK) was assigned to the putative U chromosome, and one (HSF 1) was assigned to the putative V chromosome.

**Table 2:** DhT proteins with coverage differences among the sexes in *Marchantia inflexa*.

Protein	Log_2_ (male coverage/female coverage)
Heat shock factor 1 (HSF 1)	14.3397
Calcium dependent protein kinase (CDPK)	−9.7936
Glucose related protein 94 (GRP94)	−16.4576
Aldehyde dehydrogenase (ALDH)	−4.2169
Heat shock protein 70 (HSP101)	−7.318
Heat shock protein 101 (HSP70)	−16.4576
Superoxide dismutase (SOD)	−4.095

We designated a log_2_ fold change > |4| as the cutoff to define a significant coverage difference. Negative log_2_ coverage ratios indicate higher female coverage relative to males, whereas positive log_2_ coverage ratios indicate higher male coverage.

**Figure 5:**
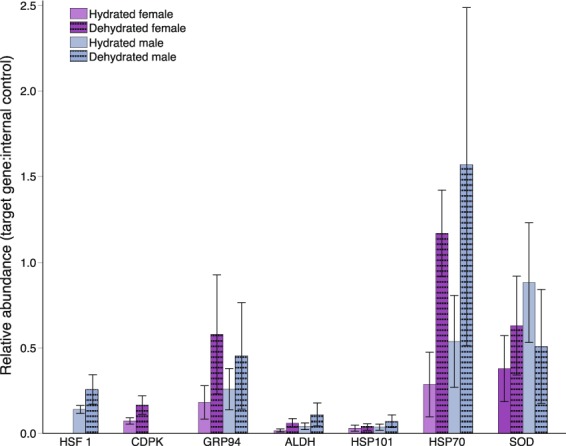
Relative expression of candidate dehydration associated genes in three male and three female plants under hydrated and dehydrated conditions. Gene expression estimates were standardized using an internal control gene (actin). The expression of each candidate gene relative to actin was computed to estimate relative abundance. Absence of a bar (as seen for HSF 1 and CDPK) indicates that there was no amplification of that gene in the corresponding sample. Error bars are standard error of the mean.

To verify that candidate DT associated genes were expressed during dehydration and to test for sex-specific patterns of expression, we conducted qPCR validation of seven candidate dehydration associated genes. We quantified expression for each gene in three males and three females under both hydrated and dehydrated conditions by qPCR. These analyses revealed that all candidate genes were expressed in *M*. *inflexa* under both hydrated and dehydrated conditions (Fig. 5). Analysis of changes in relative expression of DhT associated genes during dehydration identified an overall increase in expression during dehydration (F_1,1_ = 5.70, p = 0.019), differences among genes (F_6,6_ = 9.03, p < 0.001) and a significant interaction between gene and hydration state (F_6,6_ = 2.41, p = 0.0351). The interaction effect was driven primarily by an increase in HSP70 expression during dehydration (other genes did not show significant changes in expression during dehydration). Interestingly, the two candidate genes that were putatively sex-linked (female-specific CDPK and male-specific HSF 1) exhibited sex-specific expression, but autosomal candidate DhT genes (GRP94, ALDH, HSP101, HSP70, and SOD) were expressed at similar levels in males and females suggesting copies are present in both sexes. These autosomal genes were present at different copy number in the male and female used for genome assembly, but in the larger panel assayed by qPCR this did not translate to differences in relative expression among the sexes.

## Discussion

Our assembly of the *M*. *inflexa* genome represents a new resource for comparative studies among land plants. We capitalized on the recently published genome of related liverwort *M*. *polymorpha*^2^ to improve scaffolding of our assembly, estimate divergence rates among specific sequence types, and to identify sex-linked sequences that were leveraged to generate male and female genetic sex markers for *M*. *inflexa*. Our analyses identify several genes on the autosomes, organelles, and sex chromosomes that show strong signatures of recent diversifying selection in *Marchantia*. Additionally, we identified multiple genes possibly underlying an observed genotype difference in DhT in *M*. *inflexa*, which point towards a complex mechanism of heightened DhT. We detected differences in copy number of DhT genes across multiple loci, two of which were putatively sex-linked. Evidence of sex-linked genes underlying differences in DhT is intriguing, as prior studies indicate complex patterns of sexual dimorphism in DhT in *M*. *inflexa*^26,63^.

Analyses of dN/dS ratios for genes on autosomes, sex chromosomes, and organelles in *M*. *inflexa* and *M*. *polymorpha* showed evidence of increased diversification of sex-linked genes relative to autosomal genes, and conservation of organellar genes (particularly the chloroplast) relative to autosomes. UV sex determination systems are expected to differ from diploid dominant (XY and ZW) sex determination systems in multiple ways, due primarily to haploid selection^35,37^. However, both empirical and theoretical studies on sex chromosome evolution in haploid dominant systems are limited. Some UV sex chromosomes exhibit suppressed recombination similar to XY and ZW sex chromosomes, which can lead to degeneration of UV chromosomes^36,37^. However, because UV chromosomes are fully exposed to selection during the majority of the organism’s life cycle, positive adaptations may sweep through populations more rapidly than in XY or and ZW systems. Our analyses indicate that rapid diversification of sex-linked genes is occurring in *Marchantia*, suggesting that exposure to haploid selection can be a diversifying force on sex chromosomes.

Interestingly, the finding that *M*. *inflexa* and *M*. *polymorpha* sex-linked genes are more divergent than autosomal genes is driven primarily by diversification of male-specific (V) genes. Diversification of female-specific (U) genes, and genes with alleles on both the U and V chromosomes is similar to diversification of autosomal genes (Fig. 3). It has been suggested that UV chromosomes can become highly differentiated between the sexes due to sex-specific selection^36^. Males are generally thought to be under stronger sexual selection than females, and variability in male reproductive success may facilitate rapid adaptation of V-linked genes^35^. Further because male gametes are broadcast indiscriminately, we expect strong selection on sperm characteristics to maintain species recognition in this system^62^. Evidence for diversification of multiple male-specific genes provides a possible mechanism for maintaining species boundaries.

Sex-linked genes with high dN/dS appear to be involved in multiple cellular processes, some of which contribute to differences in sex function in other systems. Notably, the female-allele of CCR4-NOT transcription related complex (dN/dS = 4.27) is homologous to the *rcd1*^+^ gene of *Schizosaccharomyces pombe*, which is critical for nitrogen starvation induced sexual reproduction^68^. Divergence of these genes within *Marchantia* points towards specific reproductive differences among these species, which may be related to selection for species-specific recognition, habitat-specific optimization, or the evolutionary dynamics of haploid selection on sex chromosomes.

Our analyses define male and female-specific genetic sex markers, which will be of great utility in future studies of *M*. *inflexa* populations where plants are latent to express sex. The ability to sex plants via a simple PCR assay will expedite general efforts to characterize individuals and populations of *M*. *inflexa*^63^, and will aid in efforts to develop *M*. *inflexa* as a model system to investigate population dynamics and ecological genetics.

In our efforts to identify genes underlying DhT we capitalized on a previously identified genotype difference in DhT in *M*. *inflexa*^26^, targeting genes that exhibited substantial coverage differences among genotypes. We identified several such genes, which we speculated contribute to differences in DhT among genotypes. Interestingly two of these genes were putatively sex-linked, suggesting that there may be sex-specific components to DhT in *M*. *inflexa*. Importantly, qPCR validation of seven candidate dehydration associated genes in additional male and female plants revealed persistent expression of these genes under hydrated and dehydrated conditions, supporting a possible role for these genes in DhT. The unique biology of bryophytes makes them prone to rapid drying and thus, many genes involved in DhT are expressed prior to dehydration in bryophytes^43^. Various patterns of expression have been observed in DhT and DT bryophytes, including constitutive expression, expression induced during drying, and the sequestration of mRNAs in ribonucleoprotein particles^69,70^. Thus, there is no absolute predication for how a given gene will be expressed during dehydration. Our qPCR analyses suggest that even in this small set of candidate DhT genes, multiple patterns of expression are evident in *M*. *inflexa*. The majority of tested genes were expressed constitutively, but one (HSP70) showed a significant increase in expression during dehydration. Importantly, the two genes that were putatively sex-linked (female-specific CDPK and male-specific HSF1) were expressed in a strictly sex-specific pattern. Interestingly CDPKs have been recognized as important hubs in plant stress signaling pathways^71^ with highly conserved structure^72^, which provides a possible explanation for elevated DhT in females. Other candidate DhT genes were expressed at similar levels across the sexes, suggesting that despite possible involvement in DhT these genes are unlikely to drive sex-related differences in DhT within *M*. *inflexa*. Quantification of candidate DhT gene expression by qPCR further reinforces gene annotations in demonstrating that predicted *M*. *inflexa* genes are expressed in the expected tissues and individuals.

In summary, the draft genome for *M*. *inflexa* adds to a growing body of genomic resources for land plants, which will enable investigation of early plant evolution and physiology. We leveraged this assembly to identify genes under diversifying selection in *Marchantia*, to develop genetic sex markers, and to target genes contributing to DhT. Our analyses comprise one of the few empirical studies on haploid sex chromosome evolution and suggest that several sex-linked genes (particularly male-specific (V) genes) have undergone rapid diversification in *Marchantia*. We identified multiple sex-specific sequences, which were used to develop genetic sex markers and identify genes underlying differences in DhT of *M*. *inflexa*. We find evidence that DhT in *M*. *inflexa* is likely impacted by the constitutive expression of select DhT genes, and that sex differences in DhT may be impacted by sex-linked DhT genes.

## Methods

### Plant growth, DNA extraction, and sequencing

Plant specimens for genome sequencing were collected from East Turure stream (10°41′04″N 61°09′39″W) on the island of Trinidad, Republic of Trinidad and Tobago in 2009. Voucher specimens are deposited at the Missouri Botanical Garden (St. Louis, MO, USA, specimen numbers M092113 and M092115) and at the National Herbarium of the Republic of Trinidad and Tobago (St. Augustine, Trinidad, specimen number TRIN34616, D. N. McLetchie, collector). Vegetative tissue was transported to Lexington, Kentucky, USA and thirty-six clones (generated though vegetative propagation) of one male and one female genotype were planted on steam-sterilized soil and maintained in a randomized layout in a climate-controlled greenhouse. Plants were watered daily with distilled water and kept under shade cloth to mimic field conditions. Vegetative tissue (growing aerially with no soil contact) was collected from male and female plants after ~5 years in greenhouse conditions. Prior to DNA extraction, thalli were washed in distilled water three times to remove surface contamination. DNA was extracted following a CTAB extraction protocol modified from Doyle^73^. Sequencing libraries were constructed with 300 base pair (bp) inserts and whole genome sequencing was conducted on an Illumina HiSeq2000 for 100 bp paired end (PE) reads at the Beaty Biodiversity Research Centre, University of British Columbia.

### Genome assembly and annotation

Sequence read quality was assessed with fastQC version 3^74^, and filtered with Trimmomatic version 0.33^75^. Male and female reads were combined to increase coverage and k-mer plots were generated with DSK version 1.1 (see Supplementary Fig. S1)^76^. Assembly was carried out using SOAP *de novo* version 2.04-r240^77^ with a k-mer length of 31. Reads shorter than 100 bp were not included, alignments of less than 32 bp were not considered reliable, and k-mers observed nine or fewer times were excluded from the assembly.

Following initial assembly, we plotted the length and GC content of each scaffold in JMP®, Version 12 (SAS Institute Inc.). The plot revealed two distinct clusters of well-assembled (long) scaffolds: one with a mean GC content of ~65% and one with a mean GC content of ~45% (see Supplementary Fig. S3). Consequently, we probed each distinct GC cluster to identify the taxonomic source of the contributing sequence reads by aligning the 100 longest scaffolds of each GC cluster to NCBI’s refseq database^78^ using TBLASTX^79^. Taxonomic classification of the resulting alignments with Megan version 4^80^ revealed that scaffolds with high GC content were derived from a diverse microbial community, whereas scaffolds with low GC content were derived exclusively from plant material (see Supplementary Fig. S4). Notably, other members of *Marchantia* have a GC content of ~45%^81^, providing additional support for the assumption that low GC content reads were derived from *M*. *inflexa*. Consequently, we filtered the raw sequence data to remove all reads with a GC content >55%. The remaining reads (although likely not entirely contamination free) represent a data set enriched for *M*. *inflexa* genomic information. Using only reads with a GC content <55%, we reassembled the sequence data with the same parameters as above.

The resulting scaffolds were aligned to the *M*. *polymorpha* reference genome^2^ with BLASTN, allowing us to organize *M*. *inflexa* scaffolds with Chromosomer version 1.3^82^, which leverages pairwise sequence alignments and local synteny to assign orthologous regions to super-scaffolds. All *M*. *inflexa* scaffolds not mapped to *M*. *polymorpha* were appended to the super-scaffold assembly, and all contigs under 1000 bp were removed. Assembly statistics were computed using assemblathon_stats_2.pl script (https://github.com/lexnederbragt/denovo-assembly-tutorial/blob/master/scripts/assemblathon_stats_2.pl). To estimate assembly completeness, we quantified the percentage of Universal Single-copy Orthologs from the plant set of OrthoDB v9 of BUSCO v3^64^ present in the *M*. *inflexa* genome assembly. We conducted a parallel assessment of the *M*. *polymorpha* genome assembly.

To assemble the mitochondrial and chloroplast genomes of *M*. *inflexa* raw reads were trimmed with Trimmomatic version 0.33^75^ and error corrected using the ErrorCorrectReads module of Allpaths-LG version 50156^83^. These were aligned to the *M*. *polymorpha* reference plastid and mitochondrial sequences (GI 11466673^84^ and GI 11467097^11^, respectively) with BWA mem version 0.7.12-r1039^85^. Reads with alignments, plus their mates, were extracted and partitioned. Each partition was assembled separately with Ray de novo version 2.3.1^86^ at k = 31. Ray contigs were scaffolded against their homologous *M*. *polymorpha* reference sequences using Abacas version 1.3.1^87^. Adjacent contig overlaps were identified using BLASTN^88^ and merged at the shared substring. Pilon version 1.13^89^ was then run for thirty iterations. Assemblies were annotated with Daisie Huang’s script PLANN (https://github.com/daisieh/plann) and visualized in OrganellarGenomeDRAW (http://ogdraw.mpimp-golm.mpg.de/cgi-bin/ogdraw.pl).

We used Crossmap version 2.7^90^ to transfer all *M*. *polymorpha* gene annotations to orthologous *M*. *inflexa* sequences. In addition to these lift-over annotations, *de novo* gene prediction was carried out via Maker version 2.31.8^91^ for four iterations of gene finding. The resulting predicted proteins were aligned to the *M*. *polymorpha* genome^2^, *Physcometrella patens* genome^7^, the *Arabidopsis thaliana* genome^92^, and NCBI’s refseq database^78^ using BLASTP^79^. All alignments were merged and the single best hit for each *M*. *inflexa* predicted protein was selected based on bitscore. The combined set of *de novo* and lift-over annotations was used for downstream analyses.

### Sequence similarity between *M*. *inflexa* and *M*. *polymorpha*

To enable comparison of orthologous sequences, we aligned the entire *M*. *inflexa* assembly to the *M*. *polymorpha* assembly v3.1 with LASTZ version 1.04^93^, and extracted orthologous CDS, introns, and intergenic sequences from both assemblies using a combination of BEDtools version 2.19.1^94^ and BEDOPS version 2.4.35^95^. To explicitly test for differences in nucleotide differentiation among these sequence types, CDS, introns, and intergenic sequences were realigned to one another using LASTZ version 1.04. The resulting mean % identity (%ID) for each sequence type was computed and differences among sequence types were tested for significance with a mixed effects linear model in JMP®, Version 12 (SAS Institute Inc.). The fixed effect of sequence type on %ID was tested (sequence length was included in the model as a random effect).

To investigate patterns of gene divergence we computed dN/dS for all orthologous CDS in *M*. *inflexa* and *M*. *polymorpha*. Initially, we extracted the complete CDS and translated amino acid sequence for all orthologous genes using gffread (https://github.com/gpertea/gffread). Orthologous translated CDS were aligned with Clustal Omega version 1.2.4^96^, and codon aware DNA alignments were defined using PAL2NAL version 14^97^, during which all gaps and internal stop codons were removed. Next, dN/dS ratios for each ortholog were calculated with the yn00 function of PAML version 4.9^98^, which computes dN/dS using pairwise comparisons accounting for both transition/transversion bias and base/codon frequency bias^99^. Following filtering conventions^100^, cases in which dS = 0, dN >2, and dN/dS >10 were removed from the output, and dN/dS values were log transformed to satisfy assumptions of normality for statistical testing. Differences among groups in mean dN/dS were tested for significance using a mixed effects linear model in JMP®, Version 12 (SAS Institute Inc.). Initially, the fixed effect of gene type (autosomal, sex-linked, or organellar) on dN/dS was tested (scaffold ID was included in the model as a random effect). Post hoc comparisions among gene types were made using orthogonal contrasts to explicitly compare autosomal genes to sex and organallar genes. Subesquntly, we made more detailed comparisions among specific gene types with a mixed effects linear model testing the fixed effect of specific gene type (autosomal, male-specific, female-specific, male-allele, female-allele, mitochondria, and chloroplast) on dN/dS (scaffold ID was included in the model as a random effect). Again, post hoc comparisons were made using orthogonal contrasts to specifically compare each gene type to the autosomal genes. Finally, all individual genes with dN/dS values >1 were identified, and gene ontology (GO) terms were defined with the GORetreiver tool and summarized with the GOSlimViewer tool available at AgBase (http://agbase.msstate.edu/cgi-bin/tools.pl)^101^.

### Sex marker identification

Read coverage was computed using DifCover (https://github.com/timnat/DifCover)^65^ to identify regions of the genome unique to these male and female genotypes. Briefly, we determined the genotype-specific coverage by mapping male and female sequence reads back to the draft assembly with Bowtie2^102^. Coverage was calculated for 500 bp windows with BEDtools version 2.19.1^94^, and DifCover was used to calculate the log_2_ ratios of male:female coverage for each 500 bp window. *Marchantia inflexa* sequences that were both homologous to the *M*. *polymorpha* sex chromosomes and showed genotype-specific coverage were flagged as potential sex markers. PCR primers were designed with primer3^103^ for five candidate male markers and three candidate female markers.

Candidate sex markers were tested for fidelity by PCR analysis using plants from five distinct populations in Trinidad in 2016 (see Supplementary Table S3 for sample collection info). Plants were cultivated in greenhouse conditions on steam-sterilized soil, under shade cloth, and watered daily for ~one year. When plants began to produce sex organs naturally, vegetative tissue (visibly connected to a reproductive structure to ensure accurate sex identification) was collected from nine individuals of each sex. DNA was extracted from plant tissues following a modified CTAB extraction protocol (same as above), and PCR reactions were conducted with a DNA template concentration of 0.8 ng/ul and combined forward and reverse primer concentration of 0.4 uM. Reaction conditions consisted of initial denaturation at 94 °C for five minutes, followed by 34 cycles of 94 °C for 30 seconds, 60 °C for 30 seconds, 72 °C for 15 seconds, and a final extension at 72 °C for five minutes.

### Dehydration tolerance

To address specific hypotheses on DhT we probed *M*. *inflexa* and *M*. *polymorpha* annotated proteins for orthologs to a list of 195 DT genes (compiled from publicly available mRNA sequences of genes expressed under water stress in model DT plants (see Supplementary Table S2)). DT orthologs were identified using BLASTX^79^, and the single best hits for each *M*. *inflexa* and *M*. *polymorpha* sequence were determined based on bitscore. Presuming that some DT genes would be multi-copy in *M*. *inflexa*, we calculated the genotype-specific coverage of each DhT ortholog using DifCover^65^ with the aim of detecting genes contributing to the observed genotype difference in DhT in *M*. *inflexa*^26^. We targeted sequences corresponding to DhT genes showing log_2_ coverage ratios > |4| as potential contributors to the observed difference in DhT. Finally, we computed dN/dS ratios for all DhT genes and identified those exhibiting signs of diversifying selection (dN/dS > 1).

To verify that candidate dehydration associated genes were expressed during dehydration and to test for sex-specific patterns of expression, we conducted qPCR validation of seven candidate genes. To do so, we selected three male and three female plants from East Turure stream (see Supplementary Table S3). Plants were cultivated in a common garden for ~eight years prior to being subjected to dehydration treatment as described in^26^. Briefly, thallus tissues (~5 mm × 7 mm) were harvested from greenhouse cultivated plants, fully hydrated for 24 hours, and placed into desiccation chambers with an internal relative humidity (RH) of 75%. Thallus tips were randomized in Petri dishes within in the desiccation chamber. Each desiccation chamber contained 18 thallus tips (three tips from each of the six genotypes). Air circulation was maintained by inserting a small fan in the chamber, and RH was verified with a RH sensor integrated into HOBO**™** humidity sensor attached to a data logger (Onset Computer Corporation, Bourne, MA, USA). The chamber was maintained at 14 °C for 22 hours. Dehydration assays were conducted at designated times of day to reduce off target variation due to fluctuations in light, temperature, and circadian rhythms.

Plants were sampled at two time points during the dehydration assay: initial hydrated conditions and dehydrated conditions (22 hours). At each time point, tissues were removed from the desiccation chamber and immediately flash frozen in liquid nitrogen to prevent further transcriptional changes, RNA was extracted from samples using the Triazol^®^Reagent according to the manufacturer’s instructions, mRNA was isolated through poly(A) enrichment with the NEBNext® Poly(A) mRNA Magnetic Isolation Module, and cDNA synthesis was conducted with SMARTScribe™ Reverse Transcriptase using random primers. Biological replicates (genotypes) were processed independently and randomized for all downstream processing and analyses to minimize possible batch effects.

We measured gene expression for seven dehydration associated genes (GRP94, ALDH, HSP101, HSP70, SOD, female-specific CDPK, and male-specific HSF 1) by qPCR. The housekeeping gene actin was included as an internal control. Primer pairs for each gene were designed using Primer3^103^ and are listed Supplementary Table S4. qPCR reactions were carried out in 96 well plates on a Roche LightCycler^®^ 96. The reaction mix consisted of 1ul cDNA, 0.5ul each of the forward and reverse primers (10uM), 10ul Luna® Universal qPCR Master Mix, and 8ul PCR-grade H_2_O, for a final reaction volume of 20ul. A template free control was included for each primer pair. The amplification program consisted of a 60 second preincubation at 95 °C, followed by 45 cycles of 95 °C for 5 seconds, 60 °C for 15 seconds, and 72 °C for 10 seconds. Following amplification, high resolution melting analyses was conducted, via one cycle of 95 °C for 60 seconds, 40 °C for 60 seconds, 65 °C for 1 second, and 97 °C for 1 second.

Data were analyzed using the Roche LightCycler^®^ 96 Software Version 1.1. Initially, melting curve analysis was conducted to confirm product quality, and any samples with abnormal profiles were removed from the analyses. Raw Cq (quantitation cycle) values were used to estimate cDNA concentration for the seven candidate dehydration associated genes and housekeeping gene actin (used as an internal control) (see Supplementary Fig. S6). Cq indicates the first cycle at which fluorescence could be detected (smaller Cq indicates a higher starting concentration of the transcript). Subsequently, we computed the abundance of each candidate gene relative to the internal control gene actin. These analyses were performed according to the instructions for relative quantification analyses in the Roche LightCycler^®^ 96 manual to determine the ratio of target cDNA to actin (to account for potential differences in the starting concentration of cDNA among samples) based on raw Cq values and estimated cDNA concentrations. To test for changes in expression during dehydration and investigate sex differences in DhT gene expression, the relative ratio of target cDNA to actin (internal control gene) was analyzed in JMP®, Version 12 (SAS Institute Inc.). We used a mixed effects linear model to test the fixed effects of gene, hydration state, sex, and all second and third order interaction effects on the relative ratio of target:control expression. Contrasts were used to test for changes in expression during dehydration of each individual gene.

## Supplementary information

Supplementary information

## Data Availability

The unprocessed sequence data associated with this study are available in GenBank (accession numbers: SRR7348360 and SRR7348361). This Whole Genome Shotgun project (M_inflexa_v1.1) has been deposited at DDBJ/ENA/GenBank under the accession QLSQ00000000. The version described in this paper is version QLSQ01000000. The complete mitochondrial and chloroplast sequences are available at FigShare (10.6084/m9.figshare.6639209.v1).

## References

[1] Kenrick P, Crane PR (1997). The origin and early evolution of plants on land. Nature.

[2] Bowman JL (2017). Insights into land plant evolution garnered from the Marchantia polymorpha genome. Cell.

[3] Ligrone R, Duckett JG, Renzaglia KS (2012). Major transitions in the evolution of early land plants: a bryological perspective. Ann. Bot..

[4] Wellman CH, Osterloff PL, Mohiuddin U (2003). Fragments of the earliest land plants. Nature.

[5] Morris JL (2018). The timescale of early land plant evolution. Proc. Natl. Acad. Sci. USA.

[6] Jones R. Neil, Langdon Tim (2013). The Plant Nucleus at War and Peace: Genome Organization in the Interphase Nucleus. Plant Genome Diversity Volume 2.

[7] Rensing SA (2008). The Physcomitrella genome reveals evolutionary insights into the conquest of land by plants. Science (80-.)..

[8] Shaw, A. J. *et al*. The Sphagnum Genome Project. in 167–187, 10.1016/bs.abr.2016.01.003 (2016).

[9] Szövényi P (2016). The Genome of the Model Species Anthoceros agrestis. Adv. Bot. Res..

[10] Rensing SA (2017). Why we need more non-seed plant models. New Phytol..

[11] Oda K (1992). Gene organization deduced from the complete sequence of liverwort Marchantia polymorpha mitochondrial DNA. A primitive form of plant mitochondrial genome. J. Mol. Biol..

[12] Terasawa K (2007). The Mitochondrial Genome of the Moss Physcomitrella patens Sheds New Light on Mitochondrial Evolution in Land Plants. Mol. Biol. Evol..

[13] Wang B, Xue J, Li L, Liu Y, Qiu Y-L (2009). The complete mitochondrial genome sequence of the liverwort Pleurozia purpurea reveals extremely conservative mitochondrial genome evolution in liverworts. Curr. Genet..

[14] Li L, Wang B, Liu Y, Qiu Y-L (2009). The Complete Mitochondrial Genome Sequence of the Hornwort Megaceros aenigmaticus Shows a Mixed Mode of Conservative Yet Dynamic Evolution in Early Land Plant Mitochondrial Genomes. J. Mol. Evol..

[15] Myszczyński K (2017). The complete mitochondrial genome of the cryptic species C of Aneura pinguis. Mitochondrial DNA Part A.

[16] Sugiura C, Kobayashi Y, Aoki S, Sugita C, Sugita M (2003). Complete chloroplast DNA sequence of the moss Physcomitrella patens: evidence for the loss and relocation of rpoA from the chloroplast to the nucleus. Nucleic Acids Res..

[17] Kugita M, Yamamoto Y, Fujikawa T, Matsumoto T, Yoshinaga K (2003). RNA editing in hornwort chloroplasts makes more than half the genes functional. Nucleic Acids Res..

[18] Wolf PG (2005). The first complete chloroplast genome sequence of a lycophyte, Huperzia lucidula (Lycopodiaceae). Gene.

[19] Ohyama K (1996). Chloroplast and Mitochondrial Genomes from a Liverwort, Marchantia polymorpha —Gene Organization and Molecular Evolution—. Biosci. Biotechnol. Biochem..

[20] Bischler, H. & Marchantia, L. *The New World Species (Bryophytorum Bibliotheca*, *Band* 2*6)*. (Lubrecht & Cramer Ltd, 1984).

[21] Kumar S, Stecher G, Suleski M, Hedges SB (2017). TimeTree: A Resource for Timelines, Timetrees, and Divergence Times. Mol. Biol. Evol..

[22] McLetchie DN, Puterbaugh MN (2000). Population sex ratios, sex-specific clonal traits and tradeoffs among these traits in the liverwort Marchantia inflexa. Oikos.

[23] Brzyski JR, Taylor W, McLetchie DN (2014). Reproductive allocation between the sexes, across natural and novel habitats, and its impact on genetic diversity. Evol. Ecol..

[24] McLetchie DN, García-Ramos G (2017). A predictive relationship between population and genetic sex ratios in clonal species. Acta Oecologica.

[25] Brzyski, J. R., Stieha, C. R. & McLetchie, D. N. The impact of asexual and sexual reproduction in spatial genetic structure within and between populations of the dioecious plant Marchantia inflexa (Marchantiaceae). *Ann*. *Bot*, 10.1093/aob/mcy106 (2018). 10.1093/aob/mcy106 PMC6266107 29924293

[26] Marks RA, Burton JF, McLetchie DN (2016). Sex differences and plasticity in dehydration tolerance: insight from a tropical liverwort. Ann. Bot..

[27] Stieha CR, Middleton AR, Stieha JK, Trott SH, Mcletchie DN (2014). The dispersal process of asexual propagules and the contribution to population persistence in Marchantia (Marchantiaceae). Am. J. Bot..

[28] Wyatt, R. & Anderson, L. E. Breeding systems in bryophytes. *Exp*. *Biol*. *Bryophyt*.*/Ed*. *by A*.*F*. *Dye*. *J*.*G*. *Duckett* (1984).

[29] Marks Rose A., Smith Jeramiah J., Cronk Quentin, McLetchie D. Nicholas (2017). Variation in the bacteriome of the tropical liverwort, Marchantia inflexa, between the sexes and across habitats. Symbiosis.

[30] McLetchie DN, Collins AL (2001). Identification of DNA Regions Specific to the X and Y Chromosomes in Sphaerocarpos texanus. Bryologist.

[31] Korpelainen H, Bisang I, Hedenas L, Kolehmainen J (2008). The First Sex-Specific Molecular Marker Discovered in the Moss Pseudocalliergon trifarium. J. Hered..

[32] Milewicz M (2013). & Sawicki, akub. Sex-linked markers in dioecious plants. Plant Omi..

[33] Holá E, Vesalainen T, Těšitel J, Laaka-Lindberg S (2014). Sex ratio, sex-specific pattern in vegetative growth and gemma production in an aquatic liverwort, Scapania undulata (Marchantiophyta: Scapaniaceae). Bot. J. Linn. Soc..

[34] Wann FB (1925). Some of the Factors Involved in the Sexual Reproduction of Marchantia polymorpha. Am. J. Bot..

[35] Bachtrog D (2011). Are all sex chromosomes created equal?. Trends Genet..

[36] Immler S, Otto SP (2015). The evolution of sex chromosomes in organisms with separate haploid sexes. Evolution (N. Y)..

[37] Bull JJ (1978). Sex Chromosomes in Haploid Dioecy: A Unique Contrast to Muller’s Theory for Diploid Dioecy. Am. Nat..

[38] McDaniel, S. F., Neubig, K. M., Payton, A. C., Quatrano, R. S. & Cove, D. J. Recent gene-capture on the UV sex chromosomes of the moss Ceratodon purpureus. *Evolution (N. Y)*. **67** (2013). 10.1111/evo.12165 PMC3801428 24094335

[39] Rensing SA (2018). Great moments in evolution: the conquest of land by plants. Curr. Opin. Plant Biol..

[40] Turetsky MR (2003). The Role of Bryophytes in Carbon and Nitrogen Cycling. Bryologist.

[41] Shaw J (1987). Evolution of Heavy Metal Tolerance in Bryophytes. II. An Ecological and Experimental Investigation of the Copper Moss Scopelophila cataractae (Pottiaceae). Am. J. Bot..

[42] van der Wal R, Pearce ISK, Brooker RW (2005). Mosses and the struggle for light in a nitrogen-polluted world. Oecologia.

[43] Proctor MCF (2007). Desiccation-tolerance in bryophytes: a review Desiccation-tolerance in bryophytes: a review. Bryologist.

[44] Costa M-CD (2017). A footprint of desiccation tolerance in the genome of Xerophyta viscosa. Nat. Plants.

[45] Xiao L (2015). The resurrection genome of Boea hygrometrica: A blueprint for survival of dehydration. Proc. Natl. Acad. Sci..

[46] VanBuren R (2015). Single-molecule sequencing of the desiccation-tolerant grass Oropetium thomaeum. Nature.

[47] VanBuren R (2018). Extreme haplotype variation in the desiccation-tolerant clubmoss Selaginella lepidophylla. Nat. Commun..

[48] Gao B (2015). De novo transcriptome characterization and gene expression profiling of the desiccation tolerant moss Bryum argenteum following rehydration. BMC Genomics.

[49] Gao B (2017). Desiccation tolerance in bryophytes: The dehydration and rehydration transcriptomes in the desiccation-tolerant bryophyte Bryum argenteum. Sci. Rep..

[50] Gao B, Zhang D, Li X, Yang H, Wood AJ (2014). De novo assembly and characterization of the transcriptome in the desiccation-tolerant moss Syntrichia caninervis. BMC Res. Notes.

[51] Yobi A (2017). Sporobolus stapfianus: Insights into desiccation tolerance in the resurrection grasses from linking transcriptomics to metabolomics. BMC Plant Biol. 2017 171.

[52] Oliver MJ, Dowd SE, Zaragoza J, Mauget SA, Payton PR (2004). The rehydration transcriptome of the desiccation-tolerant bryophyte Tortula ruralis: transcript classification and analysis. BMC Genomics.

[53] Rodriguez MCS (2010). Transcriptomes of the desiccation-tolerant resurrection plant Craterostigma plantagineum. Plant J..

[54] Gechev TS, Dinakar C, Benina M, Toneva V, Bartels D (2012). Molecular mechanisms of desiccation tolerance in resurrection plants. Cellular and Molecular Life Sciences.

[55] Ma, C. *et al*. Transcriptomic analysis reveals numerous diverse protein kinases and transcription factors involved in desiccation tolerance in the resurrection plant Myrothamnus flabellifolia. *Hortic*. *Res*. **2**, 10.1038/hortres.2015.34 (2015). 10.1038/hortres.2015.34 PMC4595987 26504577

[56] Gechev TS (2013). Molecular mechanisms of desiccation tolerance in the resurrection glacial relic Haberlea rhodopensis. Cell. Mol. Life Sci..

[57] Dinakar C, Bartels D (2013). Desiccation tolerance in resurrection plants: new insights from transcriptome, proteome and metabolome analysis. Front. Plant Sci..

[58] Oliver MJ, Velten J, Mishler BD (2005). Desiccation tolerance in bryophytes: a reflection of the primitive strategy for plant survival in dehydrating habitats?. Integr. Comp. Biol..

[59] Grene, R., Vasquez-Robinet, C. & Bohnert, H. J. Molecular Biology and Physiological Genomics of Dehydration Stress. in Plant Desiccation Tolerance (eds Lüttge, U., Beck, E. & Bartels, D.) 255–287 (Springer Berlin Heidelberg, 2011).

[60] Oliver, M. J., Cushman, J. C. & Koster, K. L. Dehydration tolerance in plants. in lant Stress Tolerance. Methods in Molecular Biology (Methods and Protocols) (ed. Sunkar, R.) **639**, 3–24 (Humana Press, 2010). 10.1007/978-1-60761-702-0_1 20387037

[61] Palmer JD (2000). Dynamic evolution of plant mitochondrial genomes: mobile genes and introns and highly variable mutation rates. Proc. Natl. Acad. Sci. USA.

[62] Palumbi SR (1999). All males are not created equal: fertility differences depend on gamete recognition polymorphisms in sea urchins. Proc. Natl. Acad. Sci. USA.

[63] Marks, R. A., Pike, B. & Nicholas Mcletchie, D. Genetic differences in water stress tolerance track environmental exposure and exhibit a fluctuating sexual dimorphism. *Rev. Oecologia*. 10.1007/s00442-019-04538-2 31664577

[64] Simão FA, Waterhouse RM, Ioannidis P, Kriventseva EV, Zdobnov EM (2015). BUSCO: assessing genome assembly and annotation completeness with single-copy orthologs. Bioinformatics.

[65] Smith JJ (2018). The sea lamprey germline genome provides insights into programmed genome rearrangement and vertebrate evolution. Nat. Genet..

[66] Hara Y (2015). Optimizing and benchmarking de novo transcriptome sequencing: from library preparation to assembly evaluation. BMC Genomics.

[67] Monte I (2018). Ligand-receptor co-evolution shaped the jasmonate pathway in land plants. Nat. Chem. Biol..

[68] Okazaki N (1998). Novel factor highly conserved among eukaryotes controls sexual development in fission yeast. Mol. Cell. Biol..

[69] Wood AJ, Oliver MJ (1999). Translational control in plant stress: the formation of messenger ribonucleoprotein particles (mRNPs) in response to desiccation of Tortula ruralis gametophytes. Plant J..

[70] Scott HB, Oliver MJ (1994). Accumulation and polysomal recruitment of transcripts in response to desiccation and rehydration of the moss Tortula ruralis. J. Exp. Bot..

[71] Schulz, P., Herde, M. & Romeis, T. Calcium-Dependent Protein Kinases: Hubs in Plant Stress Signaling and Development. *Plant Physiol*. **163** (2013). 10.1104/pp.113.222539 PMC3793034 24014579

[72] Cheng, S.-H., Willmann, M. R., Chen, H.-C. & Sheen, J. Calcium Signaling through Protein Kinases. The Arabidopsis Calcium-Dependent Protein Kinase Gene Family. *Plant Physiol*. **129** (2002). 10.1104/pp.005645 PMC1540234 12068094

[73] Doyle JJ, Doyle JL (1987). A rapid DNA isolation procedure for small quantities of fresh leaf tissue. Phytochem. Bull..

[74] Andrews, S. FastQC A Quality Control tool for High Throughput Sequence Data. (2010).

[75] Bolger AM, Lohse M, Usadel B (2014). Trimmomatic: a flexible trimmer for Illumina sequence data. Bioinformatics.

[76] Rizk G, Lavenier D, Chikhi R (2013). DSK: k-mer counting with very low memory usage. Bioinformatics.

[77] Luo R (2012). SOAPdenovo2: an empirically improved memory-efficient short-read de novo assembler. Gigascience.

[78] Pruitt KD, Tatusova T, Maglott DR (2005). NCBI Reference Sequence (RefSeq): a curated non-redundant sequence database of genomes, transcripts and proteins. Nucleic Acids Res..

[79] Altschul SF, Gish W, Miller W, Myers EW, Lipman DJ (1990). Basic local alignment search tool. J. Mol. Biol..

[80] Huson DH, Auch AF, Qi J, Schuster SC (2007). MEGAN analysis of metagenomic data. Genome Res..

[81] Sharma N, Jung C-H, Bhalla PL, Singh MB (2014). RNA Sequencing Analysis of the Gametophyte Transcriptome from the Liverwort, Marchantia polymorpha. PLoS One.

[82] Tamazian G (2016). Chromosomer: a reference-based genome arrangement tool for producing draft chromosome sequences. Gigascience.

[83] Gnerre S (2011). High-quality draft assemblies of mammalian genomes from massively parallel sequence data. Proc. Natl. Acad. Sci..

[84] Ohyama K (1988). Structure and organization of Marchantia polymorpha chloroplast genome. I. Cloning and gene identification. J. Mol. Biol..

[85] Li, H. Aligning sequence reads, clone sequences and assembly contigs with BWA-MEM. https://doi.org/arXiv:1303.3997 (2013).

[86] Boisvert S, Laviolette F, Corbeil J (2010). Ray: Simultaneous Assembly of Reads from a Mix of High-Throughput Sequencing Technologies. J. Comput. Biol..

[87] Assefa S, Keane TM, Otto TD, Newbold C, Berriman M (2009). ABACAS: algorithm-based automatic contiguation of assembled sequences. Bioinformatics.

[88] Altschul SF (1997). Gapped BLAST and PSI-BLAST: a new generation of protein database search programs. Nucleic Acids Res..

[89] Walker BJ (2014). Pilon: An Integrated Tool for Comprehensive Microbial Variant Detection and Genome Assembly Improvement. PLoS One.

[90] Zhao H (2014). CrossMap: a versatile tool for coordinate conversion between genome assemblies. Bioinformatics.

[91] Cantarel BL (2008). MAKER: an easy-to-use annotation pipeline designed for emerging model organism genomes. Genome Res..

[92] Berardini TZ (2015). The Arabidopsis information resource: Making and mining the ‘gold standard’ annotated reference plant genome. Genesis.

[93] Harris, R. S. Improved pairwise alignment of genomic DNA. (Pennsylvania State University, 2007).

[94] Quinlan AR, Hall IM (2010). BEDTools: a flexible suite of utilities for comparing genomic features. Bioinformatics.

[95] Neph S (2012). BEDOPS: high-performance genomic feature operations. Bioinformatics.

[96] Sievers F (2011). Fast, scalable generation of high-quality protein multiple sequence alignments using Clustal Omega. Mol. Syst. Biol..

[97] Suyama M, Torrents D, Bork P (2006). PAL2NAL: robust conversion of protein sequence alignments into the corresponding codon alignments. Nucleic Acids Res..

[98] Yang Z (2007). PAML 4: Phylogenetic Analysis by Maximum Likelihood. Mol. Biol. Evol..

[99] Yang Z, Nielsen R (2000). Estimating Synonymous and Nonsynonymous Substitution Rates Under Realistic Evolutionary Models. Mol. Biol. Evol..

[100] Villanueva-Cañas JL, Laurie S, Albà MM (2013). Improving genome-wide scans of positive selection by using protein isoforms of similar length. Genome Biol. Evol..

[101] McCarthy FM (2006). AgBase: a functional genomics resource for agriculture. BMC Genomics.

[102] Langmead B, Trapnell C, Pop M, Salzberg SL (2009). Ultrafast and memory-efficient alignment of short DNA sequences to the human genome. Genome Biol..

[103] Untergasser A (2012). Primer3–new capabilities and interfaces. Nucleic Acids Res..

