# Supplementary material for: Genome of the tropical plant Marchantia inflexa: implications for sex chromosome evolution and dehydration tolerance
Source: Sci Rep. 2019 Jun 19;9:8722. doi: 10.1038/s41598-019-45039-9 (PMC6584576; doi:10.1038/s41598-019-45039-9)
Supplement: Supplementary file 1 — Supplementary information [file 41598_2019_45039_MOESM1_ESM.docx]

**Supplementary Information**

**Article in *Scientific Reports***

**Genome of the tropical plant *Marchantia inflexa*: implications for sex chromosome evolution and dehydration tolerance**

Rose A. Marks Jeramiah J. Smith, Quentin Cronk, Christopher J. Grassa, D. Nicholas McLetchie

^
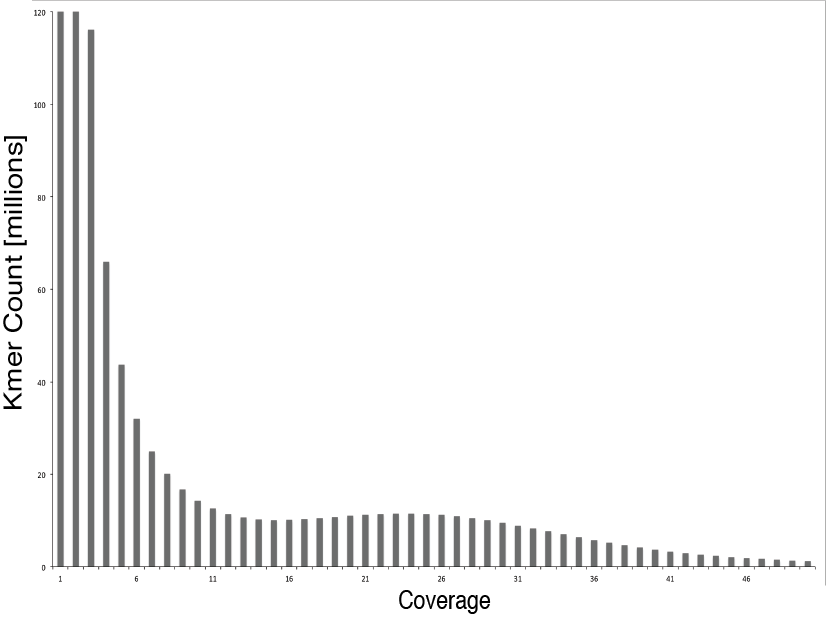
^

**Supplementary Figure S1** K-mer 31 plot of all *Marchantia inflexa* sequence reads. The estimated sequence coverage is 24x. Low frequency and unique K-mers often derive from sequencing errors or contamination. Consequently, all K-mers observed <9 times were excluded from assembly.


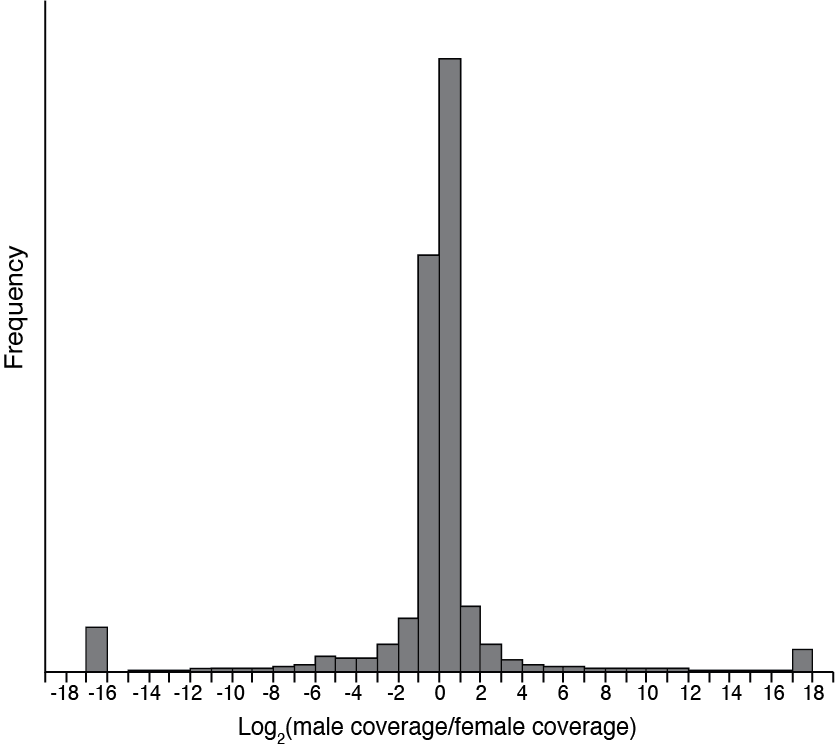


**Supplementary Figure S2** Frequency distribution of the log_2_ ratio of male:female reads mapped to the M_inflexa_v1.1 assembly. Genotype specific coverage was calculated in 500bp windows across the entire assembly. Negative ratios indicate higher female coverage, whereas positive ratios indicate higher male coverage. Sequences with 0 coverage in one sex and > 4 in the other were arbitrarily assigned to values > ± 16.

**
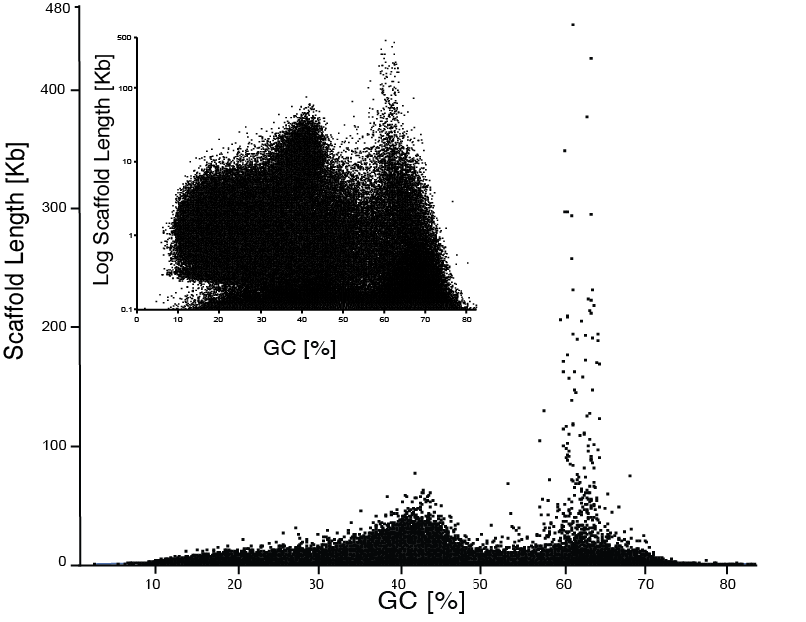
**

**Supplementary Figure S3** Plots of scaffold length (in kilobases) by GC content on both linear (main) and log_10_ (insert) scales show two distinct peaks of GC content. The low GC fraction consists of putative *Marchantia inflexa* scaffolds (other species in the genus have similarly low GC content^1^). High CG-content scaffolds are likely derived from an associated microbial community and were removed from sequence data prior to assembly of the M_inflexa_v1.1 draft genome.

**
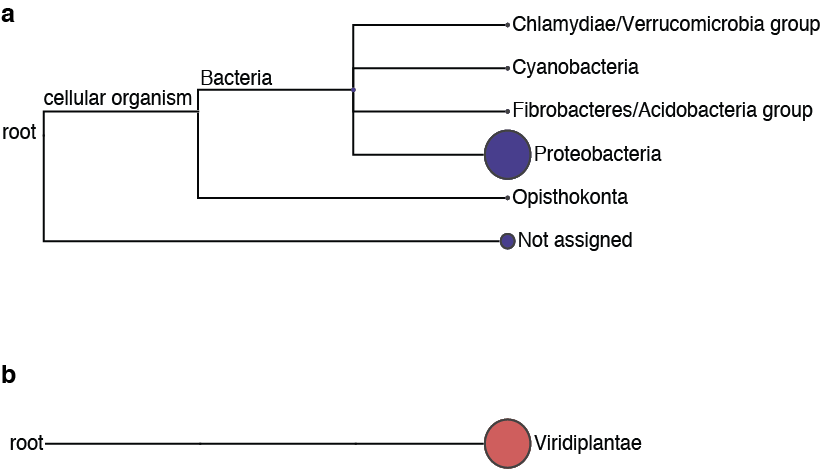
**

**Supplementary Figure S4** Taxonomic assignments of **(a)** high GC content scaffolds and **(b)** low GC content scaffolds. The size of the branch tip is proportional to the abundance of those taxa in these data.

**
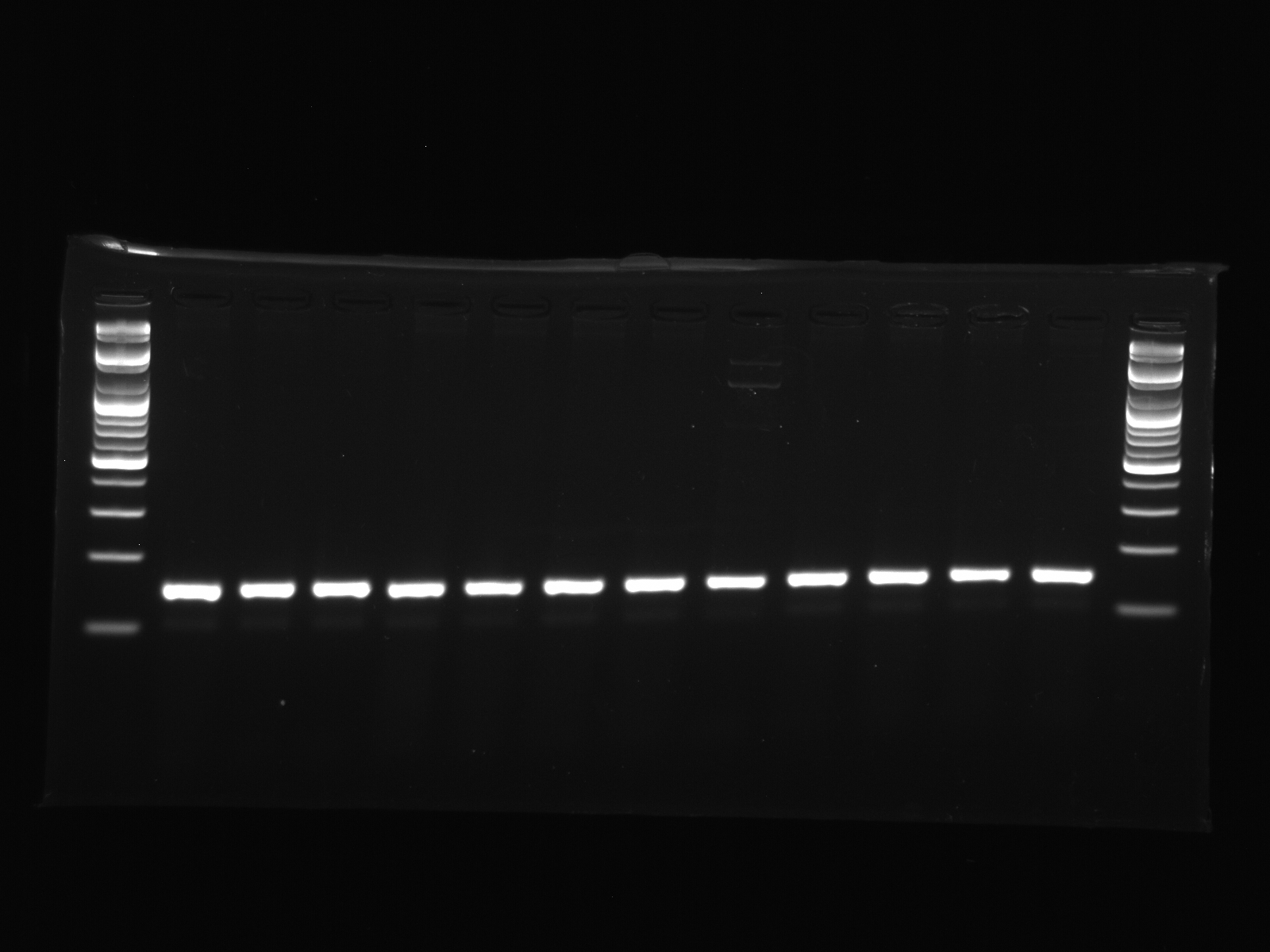

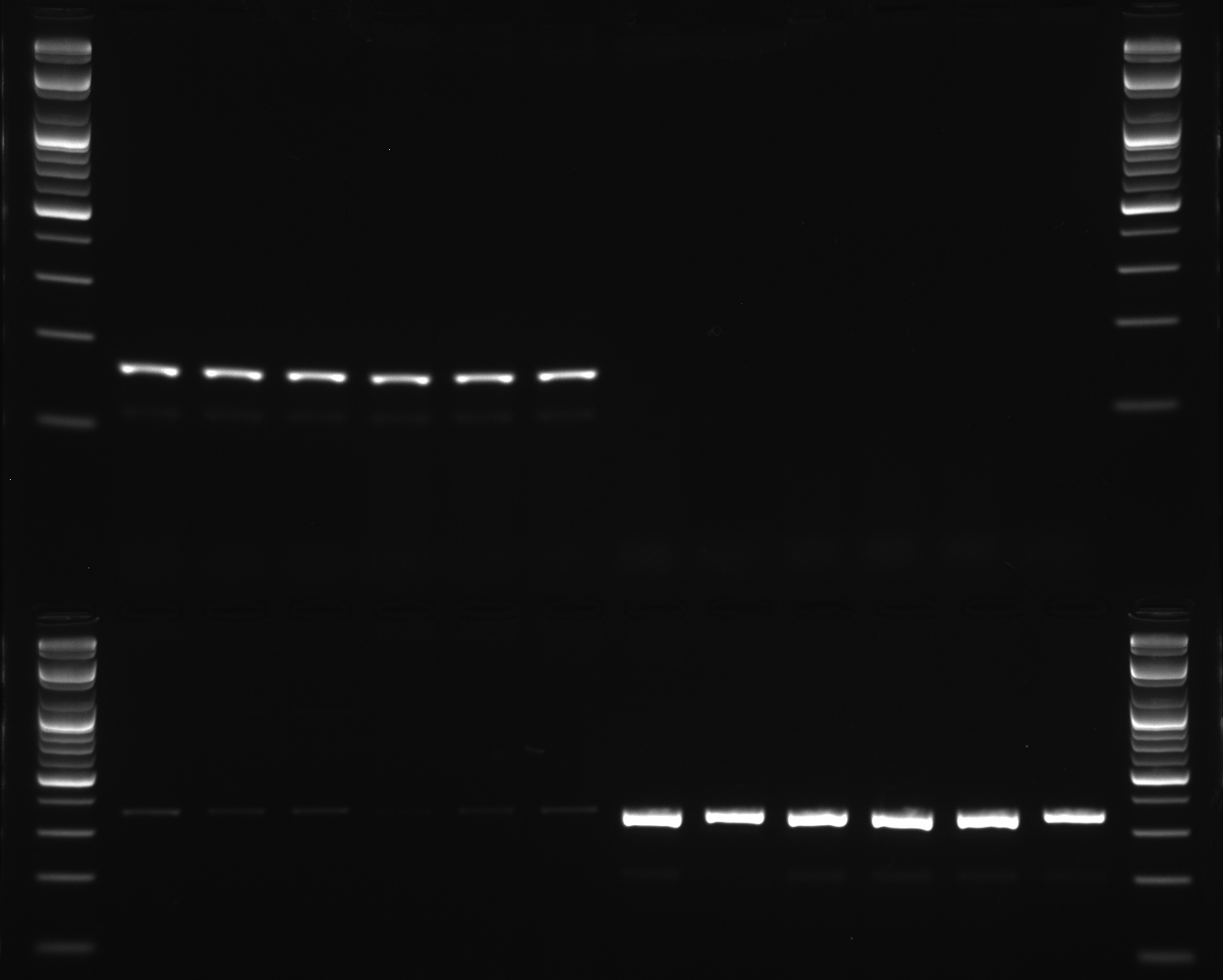
**

**Supplementary Figure S5.** Original gel images corresponding with Figure 4 in the main manuscript. The top panel is a positive control (actin), the middle panel is the male-specific sex marker, and the bottom panel is the female-specific sex marker.

**Supplementary Figure S6.** Raw Cq (quantitation cycle) values were used to estimate DNA concentration for seven candidate dehydration associated genes and housekeeping gene actin (used as an internal control). Cq was determined on a Roche LightCycler^®^ 96 and indicates the first cycle at which fluorescence could be detected (smaller Cq indicates a higher starting concentration of the transcript). Three males and three females were tested under hydrated and dehydrated conditions. Error bars are standard error of the mean, samples with a Cq of zero had no amplification of that gene.

**Supplementary Table S1.** List of all genes with dN/dS > 1. The transcript ID reported here is the ID assigned to *Marchantia polymorpha* transcripts in the v3.1 assembly available on Phytozome (<https://phytozome.jgi.doe.gov)>. Gene type refers to our categorization of genes as autosomal, sex-linked, DhT related, or mitochondrial. GO protein IDs were defined using the GOretriever tool available at AgBase (<http://www.agbase.msstate.edu)>.

| ***Transcript ID*** | ***Gene Type*** | ***dN/dS*** | ***GO protein ID*** |
| --- | --- | --- | --- |
| Mapoly0001s0022.1 | Autosomal | 1.0183 | Mechanosensitive ion channel protein 1 |
| Mapoly0003s0183.1 | Autosomal | 3.8104 | Probable carboxylesterase 18 |
| Mapoly0003s0246.1 | Autosomal | 3.8104 | Polyribonucleotide nucleotide transferase 2 |
| Mapoly0009s0052.1 | Autosomal | 3.8104 | Shewanella-like protein phosphatase 2 |
| Mapoly0009s0208.1 | Autosomal | 3.8104 | Methionine aminopeptidase 2B |
| Mapoly0010s0113.1 | Autosomal | 3.8104 | Ribosomal protein S19 |
| Mapoly0019s0040.1 | Autosomal | 3.1317 | Basic leucine zipper 61 |
| Mapoly0022s0184.1 | Autosomal | 3.1317 | Protein NRT1/ PTR FAMILY 8.2 |
| Mapoly0025s0007.1 | Autosomal | 3.1317 | kinase PAM74 |
| Mapoly0027s0071.1 | Autosomal | 3.1317 | UDP-glucuronate 4-epimerase 4 |
| Mapoly0027s0188.1 | Autosomal | 3.1317 | Inositol-pentakisphosphate 2-kinase |
| Mapoly0031s0063.1 | Autosomal | 3.1317 | Pentatricopeptide repeat-containing protein At3g06920 |
| Mapoly0031s0108.1 | Autosomal | 3.1317 | Malate dehydrogenase [NADP] |
| Mapoly0031s0181.1 | Autosomal | 3.1317 | Uncharacterized membrane protein At4g09580 |
| Mapoly0033s0148.1 | Autosomal | 2.4628 | Pathogenesis-related protein 5 |
| Mapoly0035s0037.1 | Autosomal | 2.4628 | 3-oxoacyl-[acyl-carrier-protein] synthase II |
| Mapoly0038s0053.1 | Autosomal | 2.4628 | Splicing factor U2af large subunit A |
| Mapoly0040s0067.1 | Autosomal | 2.4628 | 7-deoxyloganetin glucosyltransferase |
| Mapoly0040s0110.1 | Autosomal | 2.4628 | Kinesin-like protein KLP1 |
| Mapoly0042s0095.1 | Autosomal | 2.4628 | Glucan endo-1,3-beta-glucosidase GII |
| Mapoly0043s0098.1 | Autosomal | 2.4628 | Auxin response factor 10 |
| Mapoly0046s0122.1 | Autosomal | 2.346 | Bifunctional levopimaradiene synthase |
| Mapoly0056s0145.1 | Autosomal | 2.2798 | kinase At1g07650 |
| Mapoly0058s0080.1 | Autosomal | 2.2798 | Aldose 1-epimerase |
| Mapoly0061s0076.1 | Autosomal | 2.2798 | Pentatricopeptide repeat-containing protein At5g39980 |
| Mapoly0063s0067.1 | Autosomal | 2.2463 | Iron-sulfur assembly protein IscA |
| Mapoly0064s0048.1 | Autosomal | 1.5588 | Putative GDP-L-fucose synthase 2 |
| Mapoly0066s0022.1 | Autosomal | 1.5588 | Uncharacterized ABC transporter ATP-binding protein |
| Mapoly0067s0036.1 | Autosomal | 1.5588 | Protein SERAC1 |
| Mapoly0067s0061.1 | Autosomal | 1.5588 | Codeine O-demethylase |
| Mapoly0068s0065.1 | Autosomal | 1.5588 | Pentatricopeptide repeat-containing protein At3g18110 |
| Mapoly0071s0008.1 | Autosomal | 1.5168 | Serine/arginine-rich splicing factor RSZ23 |
| Mapoly0088s0006.1 | Autosomal | 1.0503 | Methyl-CpG-binding domain protein 4 |
| Mapoly0088s0027.1 | Autosomal | 1.0503 | Uncharacterized FAD-linked oxidoreductase YvdP |
| Mapoly0088s0041.1 | Autosomal | 1.0503 | Zinc transporter 2 |
| Mapoly0093s0044.1 | Autosomal | 1.0503 | Zinc-regulated transporter 2 |
| Mapoly0097s0084.1 | Autosomal | 1.0503 | DNA-directed RNA polymerase subunit beta' |
| Mapoly0098s0044.1 | Autosomal | 1.0503 | Exosome RNA helicase MTR4 |
| Mapoly0098s0056.1 | Autosomal | 1.0503 | Myb family transcription factor APL |
| Mapoly0106s0023.1 | Autosomal | 1.0503 | Phosphate permease PHO89 |
| Mapoly0131s0005.1 | Autosomal | 1.0503 | Patatin-like protein 2 |
| Mapoly0144s0027.1 | Autosomal | 1.0503 | Protein tas |
| Mapoly0147s0041.1 | Autosomal | 1.0503 | Alpha-N-acetylglucosaminidase |
| Mapoly0166s0010.1 | Autosomal | 1.0039 | Endochitinase CH25 |
| Mapoly0191s0007.1 | Autosomal | 1 | Ethylene-responsive transcription factor ERF110 |
| Mapoly0191s0015.1 | Autosomal | 1 | Protein-L-isoaspartate O-methyltransferase |
| Mapoly0204s0015.1 | Autosomal | 1 | Probable linoleate 9S-lipoxygenase 5 |
| Mapoly0265s0001.1 | Autosomal | 1 | Probable LRR receptor-like serine/threonine-protein |
| Mapoly0643s0001.1 | Autosomal | 1 | Probable LRR receptor-like serine/threonine-protein |
| Mapoly1175s0002.1 | Autosomal | 1 | Probable LRR receptor-like serine/threonine-protein |
| Mapoly0030s0099.1 | DhT related | 3.1317 | Aldehyde dehydrogenase family 3 member I1 |
| Mp011-91 | Mitochondria | 1.309 | orf 69 |
| Mp067-91 | Mitochondria | 1.105 | rpl 10 |
| Mp048-91 | Mitochondria | 1.0398 | orf 84 |
| Mapoly_Y_A0049 | Male | 5.107 | phosphatidylinositol-4,5-bisphosphate 3-kinase |
| Mapoly_Y_B0032 | Male | 2.3731 | Unknown function |
| Mapoly_Y_B0003 | Male | 1.1008 | Unknown function |
| Mapoly_Y_B0018 | Male allele | 1.3802 | bHLH-MYC transcription factor |
| Mapoly_0018s0021 | Female allele | 4.2679 | CCR4-NOT transcription related complex |

**Supplementary Table S2.** List of species, accession number, and the corresponding reference for all DT genes considered in our identification of *Marchantia inflexa* and *M. polymorpha* DhT orthologs.

| ***Species*** | ***Accession number*** | ***Reference*** |
| --- | --- | --- |
| *Boea hygrometrica* | AM909629.1 | ^2^ |
| *Boea hygrometrica* | AM909630.1 | ^2^ |
| *Boea hygrometrica* | EU003996.1 | ^3^ |
| *Boea hygrometrica* | EU122334.1 | ^4^ |
| *Boea hygrometrica* | EU669184.1 | ^4^ |
| *Boea hygrometrica* | FJ222452.1 | ^5^ |
| *Boea hygrometrica* | FJ222453.1 | ^5^ |
| *Boea hygrometrica* | FJ222454.1 | ^5^ |
| *Boea hygrometrica* | FJ947047.1 | ^6^ |
| *Boea hygrometrica* | GQ258824.1 | ^7^ |
| *Boea hygrometrica* | JN642717.1 | ^8^ |
| *Boea hygrometrica* | KC567294.1 | ^9^ |
| *Boea hygrometrica* | KC567295.1 | ^9^ |
| *Boea hygrometrica* | KC567296.1 | ^9^ |
| *Boea hygrometrica* | KC567297.1 | ^9^ |
| *Boea hygrometrica* | KC567298.1 | ^9^ |
| *Boea hygrometrica* | KC567299.1 | ^9^ |
| *Boea hygrometrica* | KC567300.1 | ^9^ |
| *Boea hygrometrica* | KC567301.1 | ^9^ |
| *Boea hygrometrica* | KC567302.1 | ^9^ |
| *Boea hygrometrica* | KC567303.1 | ^9^ |
| *Bryum argenteum* | KP087877.1 | ^10^ |
| *Bryum argenteum* | KP087878.1 | ^10^ |
| *Craterostigma plantagineum* | AF356001.1 | ^11^ |
| *Craterostigma plantagineum* | AF443619.1 | ^12^ |
| *Craterostigma plantagineum* | AF443620.1 | ^12^ |
| *Craterostigma plantagineum* | AF443621.1 | ^12^ |
| *Craterostigma plantagineum* | AF443622.1 | ^12^ |
| *Craterostigma plantagineum* | AF443623.1 | ^12^ |
| *Craterostigma plantagineum* | AF510112.1 | ^13^ |
| *Craterostigma plantagineum* | AJ000552.1 | ^14^ |
| *Craterostigma plantagineum* | AJ001292.1 | ^15^ |
| *Craterostigma plantagineum* | AJ001293.1 | ^15^ |
| *Craterostigma plantagineum* | AJ001294.1 | ^15^ |
| *Craterostigma plantagineum* | AJ005373.1 | ^16^ |
| *Craterostigma plantagineum* | AJ005820.1 | ^17^ |
| *Craterostigma plantagineum* | AJ005833.1 | ^17^ |
| *Craterostigma plantagineum* | AJ131999.1 | ^18^ |
| *Craterostigma plantagineum* | AJ132000.1 | ^18^ |
| *Craterostigma plantagineum* | AJ133000.1 | ^19^ |
| *Craterostigma plantagineum* | AJ133001.1 | ^19^ |
| *Craterostigma plantagineum* | AJ306960.1 | ^20^ |
| *Craterostigma plantagineum* | AY028426.1 | ^11^ |
| *Craterostigma plantagineum* | AY028427.1 | ^11^ |
| *Craterostigma plantagineum* | AY028428.1 | ^11^ |
| *Craterostigma plantagineum* | AY382595.1 | ^21^ |
| *Craterostigma plantagineum* | AY500145.1 | ^22^ |
| *Craterostigma plantagineum* | AY500146.1 | ^22^ |
| *Craterostigma plantagineum* | AY500147.1 | ^22^ |
| *Craterostigma plantagineum* | DQ073567.1 | ^23^ |
| *Craterostigma plantagineum* | DQ073569.1 | ^24^ |
| *Craterostigma plantagineum* | DQ073570.1 | ^24^ |
| *Craterostigma plantagineum* | DQ073571.1 | ^24^ |
| *Craterostigma plantagineum* | DQ073572.1 | ^24^ |
| *Craterostigma plantagineum* | DQ073573.1 | ^24^ |
| *Craterostigma plantagineum* | JQ067608.1 | ^25^ |
| *Craterostigma plantagineum* | KM377833.1 | ^26^ |
| *Craterostigma plantagineum* | KM377836.1 | ^26^ |
| *Craterostigma plantagineum* | KM377839.1 | ^26^ |
| *Craterostigma plantagineum* | KP901137.1 | ^27^ |
| *Craterostigma plantagineum* | KP901138.1 | ^27^ |
| *Craterostigma plantagineum* | KP901139.1 | ^27^ |
| *Craterostigma plantagineum* | KP901140.1 | ^27^ |
| *Craterostigma plantagineum* | KP901141.1 | ^27^ |
| *Craterostigma plantagineum* | KP901142.1 | ^27^ |
| *Craterostigma plantagineum* | KT893871.1 | ^28^ |
| *Craterostigma plantagineum* | KT893872.1 | ^28^ |
| *Craterostigma plantagineum* | KT893873.1 | ^28^ |
| *Craterostigma plantagineum* | M62987.1 | ^12^ |
| *Craterostigma plantagineum* | M62988.1 | ^12^ |
| *Craterostigma plantagineum* | M62989.1 | ^12^ |
| *Craterostigma plantagineum* | M62990.1 | ^12^ |
| *Craterostigma plantagineum* | M62991.1 | ^12^ |
| *Craterostigma plantagineum* | U33917.1 | ^29^ |
| *Craterostigma plantagineum* | X66598.1 | ^30^ |
| *Craterostigma plantagineum* | X78307.1 | ^31^ |
| *Craterostigma plantagineum* | Y11795.1 | ^32^ |
| *Craterostigma plantagineum* | Y11821.1 | ^32^ |
| *Craterostigma plantagineum* | Y11822.1 | ^32^ |
| *Craterostigma plantagineum* | Z46646.1 | ^33^ |
| *Craterostigma plantagineum* | Z46647.1 | ^33^ |
| *Craterostigma plantagineum* | Z46648.1 | ^33^ |
| *Funaria hygrometrica* | AF087640.1 | ^34^ |
| *Funaria hygrometrica* | AF089842.1 | ^34^ |
| *Funaria hygrometrica* | AF089843.1 | ^34^ |
| *Funaria hygrometrica* | AF089844.1 | ^34^ |
| *Funaria hygrometrica* | AF089845.1 | ^34^ |
| *Funaria hygrometrica* | AF089846.1 | ^34^ |
| *Funaria hygrometrica* | AF197941.1 | ^34^ |
| *Funaria hygrometrica* | AF197942.1 | ^34^ |
| *Funaria hygrometrica* | DQ239566.1 | ^35^ |
| *Haberlea rhodopensis* | HQ615875.1 | ^36^ |
| *Haberlea rhodopensis* | HQ615876.1 | ^36^ |
| *Haberlea rhodopensis* | HQ615877.1 | ^36^ |
| *Haberlea rhodopensis* | HQ634776.1 | ^36^ |
| *Haberlea rhodopensis* | HQ634777.1 | ^36^ |
| *Haberlea rhodopensis* | HQ634778.1 | ^36^ |
| *Selaginella lepidophylla* | U96715.1 | ^37^ |
| *Selaginella lepidophylla* | U96736.1 | ^37^ |
| *Sporobolus stapfianus* | AF083256.1 | ^38^ |
| *Sporobolus stapfianus* | AJ242801.1 | ^39^ |
| *Sporobolus stapfianus* | AJ242802.1 | ^39^ |
| *Sporobolus stapfianus* | AJ242803.1 | ^39^ |
| *Sporobolus stapfianus* | AJ242804.1 | ^39^ |
| *Sporobolus stapfianus* | AJ242805.1 | ^39^ |
| *Sporobolus stapfianus* | AJ242806.1 | ^39^ |
| *Sporobolus stapfianus* | AM261428.1 | ^40^ |
| *Sporobolus stapfianus* | AM268210.1 | ^40^ |
| *Sporobolus stapfianus* | AM268211.1 | ^40^ |
| *Sporobolus stapfianus* | AM268212.1 | ^40^ |
| *Syntrichia caninervis* | GQ245973.1 | ^41^ |
| *Syntrichia caninervis* | HG764415.1 | ^42^ |
| *Syntrichia caninervis* | KJ398821.1 | ^43^ |
| *Syntrichia caninervis* | KJ398822.1 | ^43^ |
| *Syntrichia caninervis* | KJ398823.1 | ^43^ |
| *Syntrichia caninervis* | KJ398824.1 | ^43^ |
| *Syntrichia caninervis* | KJ398825.1 | ^43^ |
| *Syntrichia caninervis* | KJ398826.1 | ^43^ |
| *Syntrichia caninervis* | KJ398827.1 | ^43^ |
| *Syntrichia caninervis* | KJ398828.1 | ^43^ |
| *Syntrichia caninervis* | KJ398829.1 | ^43^ |
| *Syntrichia caninervis* | KJ398830.1 | ^43^ |
| *Syntrichia caninervis* | KJ398831.1 | ^43^ |
| *Syntrichia caninervis* | KJ398832.1 | ^43^ |
| *Syntrichia caninervis* | KJ398833.1 | ^43^ |
| *Syntrichia caninervis* | KJ398834.1 | ^43^ |
| *Syntrichia caninervis* | KJ398835.1 | ^43^ |
| *Syntrichia caninervis* | KJ398836.1 | ^43^ |
| *Syntrichia caninervis* | KM363766.1 | ^42^ |
| *Syntrichia caninervis* | KM363767.1 | ^42^ |
| *Syntrichia caninervis* | KU613409.1 | ^44^ |
| *Syntrichia caninervis* | KU613410.1 | ^44^ |
| *Syntrichia caninervis* | KU613411.1 | ^44^ |
| *Syntrichia caninervis* | KU613412.1 | ^44^ |
| *Syntrichia caninervis* | KU613413.1 | ^44^ |
| *Syntrichia caninervis* | KU613414.1 | ^44^ |
| *Syntrichia caninervis* | KU613415.1 | ^44^ |
| *Syntrichia caninervis* | KU613416.1 | ^44^ |
| *Syntrichia caninervis* | KU613417.1 | ^44^ |
| *Syntrichia caninervis* | KU613418.1 | ^44^ |
| *Syntrichia ruralis* | AF093108.1 | ^45^ |
| *Syntrichia ruralis* | AF303372.1 | ^46^ |
| *Syntrichia ruralis* | AF432345.1 | ^47^ |
| *Syntrichia ruralis* | AF470350.1 | ^48^ |
| *Syntrichia ruralis* | AY034888.1 | ^49^ |
| *Syntrichia ruralis* | AY034889.1 | ^50^ |
| *Syntrichia ruralis* | AY034890.1 | ^51^ |
| *Syntrichia ruralis* | AY034891.1 | ^51^ |
| *Syntrichia ruralis* | AY168202.1 | ^52^ |
| *Syntrichia ruralis* | U21679.1 | ^53^ |
| *Syntrichia ruralis* | U40818.1 | ^53^ |
| *Syntrichia ruralis* | U82087.1 | ^54^ |
| *Xerophyta sp.* | AF133841.1 | ^55^ |
| *Xerophyta sp.* | AF484696.1 | ^56^ |
| *Xerophyta sp.* | AF544993.1 | ^57^ |
| *Xerophyta sp.* | AF545583.1 | ^58^ |
| *Xerophyta sp.* | AF545584.1 | ^58^ |
| *Xerophyta sp.* | AF545585.1 | ^58^ |
| *Xerophyta sp.* | AY100455.1 | ^59^ |
| *Xerophyta sp.* | AY146990.1 | ^60^ |
| *Xerophyta sp.* | AY186241.1 | ^61^ |
| *Xerophyta sp.* | AY266308.1 | ^62^ |
| *Xerophyta sp.* | AY323824.1 | ^63^ |
| *Xerophyta sp.* | AY462241.1 | ^64^ |
| *Xerophyta sp.* | AY566693.1 | ^61^ |
| *Xerophyta sp.* | AY566694.1 | ^61^ |
| *Xerophyta sp.* | AY566695.1 | ^61^ |
| *Xerophyta sp.* | AY566696.1 | ^61^ |
| *Xerophyta sp.* | AY566697.1 | ^61^ |
| *Xerophyta sp.* | AY566698.1 | ^61^ |
| *Xerophyta sp.* | AY570974.1 | ^61^ |
| *Xerophyta sp.* | AY570975.1 | ^61^ |
| *Xerophyta sp.* | AY570976.1 | ^61^ |
| *Xerophyta sp.* | AY570977.1 | ^61^ |
| *Xerophyta sp.* | AY570978.1 | ^61^ |
| *Xerophyta sp.* | AY570979.1 | ^61^ |
| *Xerophyta sp.* | AY611006.1 | ^61^ |
| *Xerophyta sp.* | AY695110.1 | ^61^ |
| *Xerophyta sp.* | DQ067928.1 | ^65^ |
| *Xerophyta sp.* | DQ067929.1 | ^65^ |
| *Xerophyta sp.* | DQ149581.1 | ^65^ |
| *Xerophyta sp.* | EF017945.1 | ^66^ |
| *Xerophyta sp.* | EF449773.1 | ^67^ |
| *Xerophyta sp.* | EU333003.1 | ^68^ |
| *Xerophyta sp.* | HM123762.1 | ^69^ |
| *Xerophyta sp.* | HM123763.1 | ^69^ |
| *Xerophyta sp.* | KJ664273.1 | ^70^ |
| *Xerophyta sp.* | KJ664274.1 | ^70^ |
| *Xerophyta sp.* | KJ664310.1 | ^70^ |
| *Xerophyta sp.* | KJ664340.1 | ^70^ |
| *Xerophyta sp.* | KJ664348.1 | ^70^ |
| *Xerophyta sp.* | KJ664445.1 | ^70^ |
| *Xerophyta sp.* | KU578042.1 | ^71^ |
| *Xerophyta sp.* | KU700668.1 | ^72^ |

**Supplementary Table S3** Habitat type, site name, and coordinates of the locations where *Marchantia inflexa* plants were collected in Trinidad, Republic of Trinidad and Tobago. These collections were used to validate the fidelity of *M. inflexa* genetic sex markers. Plants from East Turure stream were used for qPCR validation of dehydration associated gene expression. Plants were collected in 2016 and cultured under greenhouse conditions at the University of Kentucky, Lexington KY, USA for 18 months prior to use.

| ***Habitat type*** | ***Site name*** | ***Coordinates*** |
| --- | --- | --- |
| Stream | East Turure | 10^o^41’04”N 61^o^09’39”W |
| Stream | West Turure | 10^o^41’00”N 61^o^10’04”W |
| Stream | Quare | 10^o^40’37”N 61^o^11’40”W |
| Road | Guanapo | 10^o^41’08”N 61^o^15’49”W |
| Road | Cumaca | 10^o^41’11”N 61^o^09’45”W |

**Supplementary Table S4** Primer sequences of candidate dehydration associated genes validated by qPCR.

| ***Gene name*** | ***Left primer sequence (5' to 3')*** | ***Right primer sequence (5’ to 3’)*** |
| --- | --- | --- |
| Actin | ACCGCTCTTCCCGATCTTAG | CAGCAATATCAGGACGCCAG |
| HSF 1 | CGAAAGTCCAATCGAACCAT | CCCTTGGGTCTGAGTTGTGT |
| CDPK | AGCGCTTTTCGGAGATTGTA | ATTACTCCGGCACTCCACAC |
| GRP94 | AAGAACGATGCGGATACCAC | AAATCCGCCACTGAAGATTG |
| ALDH | TTTTTGCATCCGTTGGTACA | AATGGATCACCAGCCTTGAC |
| HSP 101 | TCCAGCAGTTCAGTCAGTGG | GGCCTTAGCTAGCTCCGTTT |
| HSP 70 | GAAGCCCATTTGGATGAGAA | GTCCCTCGACAGAGAAGTGC |
| SOD | CGTTGCAAAGTTCGACTTGA | GTTCTCGATCTGTGCGTTCA |

**References**

1. Sharma, N., Jung, C.-H., Bhalla, P. L. & Singh, M. B. RNA Sequencing Analysis of the Gametophyte Transcriptome from the Liverwort, Marchantia polymorpha. *PLoS One* **9,** e97497 (2014).

2. Yang, Z. PAML 4: Phylogenetic Analysis by Maximum Likelihood. *Mol. Biol. Evol.* **24,** 1586–1591 (2007).

3. Wang, L., Shang, H., Wu, R. & Deng, X. A cell wall localized glycine-rich protein plays a role in dehydration tolerence in the resurrection plant Boea hygrometrica. *Unpublished* www.ncbi.nlm.nih.gov (2007).

4. Liu, X. *et al.* LEA 4 group genes from the resurrection plant Boea hygrometrica confer dehydration tolerance in transgenic tobacco. *Plant Sci.* **176,** 90–98 (2009).

5. Wang, Z., Deng, X., Wang, L. & Liu, X. A dehydration-induced galactinol synthase gene from the resurrection plant Boea hygrometrica. *Unpublished* www.ncbi.nlm.nih.gov (2008).

6. Zhu, Y. *et al.* Ectopic over-expression of BhHsf1, a heat shock factor from the resurrection plant Boea hygrometrica, leads to increased thermotolerance and retarded growth in transgenic Arabidopsis and tobacco. *Plant Mol. Biol.* **71,** 451–467 (2009).

7. Wang, Z., Wu, R. H., Wang, L. L. & Deng, X. ACO gene induction and ethylene accumulation in response to dehydration are important for subsequent recovery in the resurrection plant Boea hygrometrica. *Unpublished* www.ncbi.nlm.nih.gov (2009).

8. Wang, L. *et al.* A role for a cell wall localized glycine-rich protein in dehydration and rehydration of the resurrection plant *Boea hygrometrica*. *Plant Biol.* **11,** 837–848 (2009).

9. Zhang, Z., Wang, B., Sun, D. & Deng, X. Molecular cloning and differential expression of sHSP gene family members from the resurrection plant Boea hygrometrica in response to abiotic stresses. *Unpublished* www.ncbi.nlm.nih.gov (2013).

10. Wang, M. Q. & Zhang, D. Y. Direct submission. *Unpublished* www.ncbi.nlm.nih.gov (2014).

11. Phillips, J., Hilbricht, T., Salamini, F. & Bartels, D. A novel abscisic acid- and dehydration-responsive gene family from the resurrection plant Craterostigma plantagineum encodes a plastid-targeted protein with DNA-binding activity. *Planta* **215,** 258–266 (2002).

12. Piatkowski, D., Schneider, K., Salamini, F. & Bartels, D. Characterization of Five Abscisic Acid-Responsive cDNA Clones Isolated from the Desiccation-Tolerant Plant Craterostigma plantagineum and Their Relationship to Other Water-Stress Genes1. *Plant Physiol* **94,** 1682–1688 (1990).

13. Villalobos, M. A., Bartels, D. & Iturriaga, G. Stress Tolerance and Glucose Insensitive Phenotypes in Arabidopsis Overexpressing the CpMYB10 Transcription Factor Gene. *PLANT Physiol.* **135,** 309–324 (2004).

14. Chandler, J. W. & Bartels, D. Structure and function of the vp1 gene homologue from the resurrection plant Craterostigma plantagineum Hochst. *Mol. Gen. Genet.* **256,** 539–46 (1997).

15. Mariaux, J.-B., Bockel, C., Salamini, F. & Bartels, D. Desiccation- and abscisic acid-responsive genes encoding major intrinsic proteins (MIPs) from the resurrection plant Craterostigma plantagineum. *Plant Mol. Biol.* **38,** 1089–1099 (1998).

16. Heino, P., Nylander, M., Palva, T. & Bartels, D. Isolation of a cDNA corresponding to a protein kinase differentially expressed in the resurrection plant Craterostigma plantagineum. *Unpublished* www.ncbi.nlm.nih.gov (1998).

17. Frank, W., Phillips, J., Salamini, F. & Bartels, D. Two dehydration-inducible transcripts from the resurrection plant Craterostigma plantagineum encode interacting homeodomain-leucine zipper proteins. *Plant J.* **15,** 413–21 (1998).

18. Kleines, M. *et al.* Isolation and expression analysis of two stress-responsive sucrose-synthase genes from the resurrection plant Craterostigma plantagineum (Hochst.). *Planta* **209,** 13–24 (1999).

19. Frank, W., Munnik, T., Kerkmann, K., Salamini, F. & Bartels, D. Water deficit triggers phospholipase D activity in the resurrection plant Craterostigma plantagineum. *Plant Cell* **12,** 111–24 (2000).

20. Kirch, H. H., Nair, A. & Bartels, D. Novel ABA- and dehydration-inducible aldehyde dehydrogenase genes isolated from the resurrection plant Craterostigma plantagineum and Arabidopsis thaliana. *Plant J.* **28,** 555–67 (2001).

21. Rodrigo, M. J., Bockel, C., Blervacq, A. S., Salamini, F. & Bartels, D. Molecular characterization of a novel early dehydration inducible gene Lea-like of Craterostigma plantagineum. *Unpublished* www.ncbi.nlm.nih.gov (2003).

22. Jones, L. & McQueen-Mason, S. A role for expansins in dehydration and rehydration of the resurrection plant *Craterostigma plantagineum*. *FEBS Lett.* **559,** 61–65 (2004).

23. Ditzer, A., Kirch, H.-H., Nair, A. & Bartels, D. Molecular characterization of two alanine-rich Lea genes abundantly expressed in the resurrection plant C. plantagineum in response to osmotic stress and ABA. *J. Plant Physiol.* **158,** 623–633 (2001).

24. Ditzer, A. & Bartels, D. Identification of a dehydration and ABA-responsive promoter regulon and isolation of corresponding DNA binding proteins for the group 4 LEA gene CpC2 from C. plantagineum. *Plant Mol. Biol.* **61,** 643–663 (2006).

25. Petersen, J. *et al.* The lysine-rich motif of intrinsically disordered stress protein CDeT11-24 from Craterostigma plantagineum is responsible for phosphatidic acid binding and protection of enzymes from damaging effects caused by desiccation. *J. Exp. Bot.* **63,** 4919–4929 (2012).

26. Giarola, V., Krey, S., Frerichs, A. & Bartels, D. Taxonomically restricted genes of Craterostigma plantagineum are modulated in their expression during dehydration and rehydration. *Planta* **241,** 193–208 (2015).

27. Giarola, V., Challabathula, D. & Bartels, D. Quantification of expression of dehydrin isoforms in the desiccation tolerant plant Craterostigma plantagineum using specifically designed reference genes. *Plant Sci.* **236,** 103–115 (2015).

28. Giarola, V., Krey, S., von den Driesch, B. & Bartels, D. The *Craterostigma plantagineum* glycine-rich protein CpGRP1 interacts with a cell wall-associated protein kinase 1 (CpWAK1) and accumulates in leaf cell walls during dehydration. *New Phytol.* **210,** 535–550 (2016).

29. Iturriaga, G. *et al.* A family of novel myb-related genes from the resurrection plant Craterostigma plantagineum are specifically expressed in callus and roots in response to ABA or desiccation. *Plant Mol. Biol.* **32,** 707–16 (1996).

30. Bartels, D., Hanke, C., Schneider, K., Michel, D. & Salamini, F. A desiccation-related Elip-like gene from the resurrection plant Craterostigma plantagineum is regulated by light and ABA. *EMBO J.* **11,** 2771–8 (1992).

31. Velasco, R., Salamini, F. & Bartels, D. Dehydration and ABA increase mRNA levels and enzyme activity of cytosolic GAPDH in the resurrection plant Craterostigma plantagineum. *Plant Mol. Biol.* **26,** 541–6 (1994).

32. Ingram, J., Chandler, J. W., Gallagher, L., Salamini, F. & Bartels, D. Analysis of cDNA clones encoding sucrose-phosphate synthase in relation to sugar interconversions associated with dehydration in the resurrection plant Craterostigma plantagineum Hochst. *Plant Physiol.* **115,** 113–21 (1997).

33. Bernacchia, G., Schwall, G., Lottspeich, F., Salamini, F. & Bartels, D. The transketolase gene family of the resurrection plant Craterostigma plantagineum: differential expression during the rehydration phase. *EMBO J.* **14,** 610–8 (1995).

34. Waters, E. R. & Vierling, E. The Diversification of Plant Cytosolic Small Heat Shock Proteins Preceded the Divergence of Mosses. *Mol. Biol. Evol* **16,** 127–139 (1999).

35. Walters, E. R. HSP100 homolog in the moss Funaria hygrometrica. *Unpublished* www.ncbi.nlm.nih.gov (2005).

36. Apostolova, E. *et al.* Molecular cloning and characterization of cDNAs of the superoxide dismutase gene family in the resurrection plant Haberlea rhodopensis. (2012). doi:10.1016/j.plaphy.2012.03.015

37. Choi, D.-W., Close, T. J. & Iturriaga, G. Direct submission. *Unpublished* www.ncbi.nlm.nih.gov (1997).

38. O’Mahony, P. J. & Oliver, M. J. Characterization of a desiccation-responsive small GTP-binding protein (Rab2) from the desiccation-tolerant grass Sporobolus stapfianus. *Plant Mol. Biol.* **39,** 809–21 (1999).

39. Neale, A. D. *et al.* The isolation of genes from the resurrection grass Sporobolus stapfianus which are induced during severe drought stress. *Plant, Cell Environ.* **23,** 265–277 (2000).

40. Le, T. N. *et al.* Desiccation-tolerance specific gene expression in leaf tissue of the resurrection plant Sporobolus stapfianus. *Unpublished* www.ncbi.nlm.nih.gov (2006).

41. Yang, H., Zhang, D., Wang, J., Wood, A. J. & Zhang, Y. Molecular cloning of a stress-responsive aldehyde dehydrogenase gene ScALDH21 from the desiccation-tolerant moss Syntrichia caninervis and its responses to different stresses. *Mol. Biol. Rep.* **39,** 2645–2652 (2012).

42. Gao, B. & Zhang, D. Identification and analysis of VOZ transcription factor in the moss Syntrichia caninervis. *Unpublished* www.ncbi.nlm.nih.gov (2013).

43. Li, X. *et al.* Characterization of reference genes for RT-qPCR in the desert moss Syntrichia caninervis in response to abiotic stress and desiccation/rehydration. *Front. Plant Sci.* **6,** (2015).

44. Li, H., Zhang, D., Li, X., Guan, K. & Yang, H. Novel DREB A-5 subgroup transcription factors from desert moss (Syntrichia caninervis) confers multiple abiotic stress tolerance to yeast. *J. Plant Physiol.* **194,** 45–53 (2016).

45. Duff, R. J. *et al.* The Electronic Plant Gene Register Nucleotide Sequence of a Truncated cDNA (Accession No. AF093108) Encoding a H3-Like Histone Protein from the Bryophyte Tortula ruralis. Isolation of a cDNA Clone Encoding 1-Aminocyclopropane- 1-Carboxylate Oxidase from Dendrobium crumenatum. 1–618

46. Chen, X., Kanokporn, T., Zeng, Q., Wilkins, T. A. & Wood, A. J. Characterization of the V-type H((+))-ATPase in the resurrection plant Tortula ruralis: accumulation and polysomal recruitment of the proteolipid c subunit in response to salt-stress. *J. Exp. Bot.* **53,** 225–32 (2002).

47. Chen, X. & Wood, A. J. The 26S Proteasome of the Resurrection Plant Tortula ruralis: Cloning and Characterization of the TrRPT2 Subunit. *Biol. Plant.* **46,** 363–368 (2003).

48. Wood, A. J. & Chen, X. WD40 homolog from the desiccation-tolerant moss Tortula ruralis. *Unpublished* www.ncbi.nlm.nih.gov (2002).

49. Chen, X., Zeng, Q. & Wood, A. J. The stress-responsive Tortula ruralis gene ALDH21A1 describes a novel eukaryotic aldehyde dehydrogenase protein family. *J. Plant Physiol* **159,** 677–684 (2002).

50. Chen, X., Zeng, Q. & Wood, A. J. Aldh7B6 Encodes a Turgor-Responsive Aldehyde Dehydrogenase Homologue That Is Constitutively Expressed in Tortula ruralis Gametophytes. *The Bryologist* **105,** 177–184

51. Zeng, Q., Chen, X. & Wood, A. J. Two early light-inducible protein (ELIP) cDNAs from the resurrection plant Tortula ruralis are differentially expressed in response to desiccation, rehydration, salinity, and high light. *Journal of Experimental Botany* **53,** 1197–1205 (2002).

52. Wood, A. J. & Chen, X. The Tortula ruralis cDNA TrDr3 encodes a novel membrane protein. *Unpublished* www.ncbi.nlm.nih.gov (2002).

53. Scott, H. B. & Oliver, M. J. Accumulation and polysomal recruitment of transcripts in response to desiccation and rehydration of the moss *Tortula ruralis*. *J. Exp. Bot.* **45,** 577–583 (1994).

54. McKnight, S. L., Oliver, M. J. & Putnam-Evans, C. L. The electronic Plant Gene Register. *Plant Physiol.* **115,** 313–5 (1997).

55. Mundree, S. G., Whittaker, A., Thomson, J. A. & Farrant, J. M. An aldose reductase homolog from the resurrection plant Xerophyta viscosa Baker. *Planta* **211,** 693–700 (2000).

56. Mowla, S. B., Thomson, J. A., Farrant, J. M. & Mundree, S. G. A novel stress-inducible antioxidant enzyme identified from the resurrection plant Xerophyta viscosa. *Unpublished* www.ncbi.nlm.nih.gov (2002).

57. Walford, S. A., Thomson, J. A., Farrant, J. M. & Mundree, S. G. Isolation and characterization of a novel dehydration-induced Grp94 homolog from the resurrection plant Xerophyta viscosa. *Unpublished* www.ncbi.nlm.nih.gov (2002).

58. Collett, H. Genes differentially expressed during the dehydration-rehydration cycle in Xerophyta humilis. *Unpublished* www.ncbi.nlm.nih.gov (2002).

59. Garwe, D., Mundree, S. G. & Thomson, J. . Molecular characterization of XVSAP1, a stress-responsive gene isolated from the resurrection plant Xerophyta viscosa Baker. *Unpublished* www.ncbi.nlm.nih.gov (2002).

60. Butowt, R. & Collett, H. PsbR is differentially expressed during the dehydration-rehydration cycle in Xerophyta humilis. *Unpublished* www.ncbi.nlm.nih.gov (2002).

61. Collett, H. *et al.* Towards transcript profiling of desiccation tolerance in Xerophyta humilis: Construction of a normalized 11 k X. humilis cDNA set and microarray expression analysis of 424 cDNAs in response to dehydration. *Physiol. Plant.* **122,** 39–53 (2004).

62. Baker, B., Mundree, S. G. & Thomson, J. A. Xerophyta viscosa Dehydrin, Drought-Induced Protein. *Unpublished* www.ncbi.nlm.nih.gov (2003).

63. Majee, M., Majumder, A. N. L. & Mundree, S. G. Molecular characterization of XvIno1, a myo-inositol-1-phosphate synthase from the resurrection plant Xerophyta viscosa. *Unpublished* www.ncbi.nlm.nih.gov (2003).

64. Marais, S., Thomson, J. A., Farrant, J. M. & Mundree, S. G. XvVHA-c’’1-a novel stress responsive V-ATPase subunit c’’ homolog isolated from the resurrection plant Xerophyta viscosa Baker. *Unpublished* www.ncbi.nlm.nih.gov (2003).

65. Collett, H. Direct submission. *Unpublished* www.ncbi.nlm.nih.gov (2005).

66. Peters, S., Mundree, S. G., Thomson, J. A., Farrant, J. M. & Keller, F. Protection mechanisms in the resurrection plant Xerophyta viscosa (Baker): both sucrose and raffinose family oligosaccharides (RFOs) accumulate in leaves in response to water deficit. *J. Exp. Bot.* **58,** 1947–1956 (2007).

67. Chopera, D. R., Peters, S. & Keller, F. Protection mechanisms in the resurrection plant Xerophyta viscosa: Cloning, expression, characterisation and role of XvINO1, a gene coding for a myo-inositol 1-phosphate synthase. (2008). doi:10.1071/FP07142

68. Govender, K., Thomson, J. A. & Mundree, S. G. The resurrection plant Xerophyta viscosa (Baker) expresses a novel type II peroxiredoxin (XvPrx2) containing a single catalytic cysteine. *Unpublished* www.ncbi.nlm.nih.gov (2007).

69. McDonald, Z. E. The role of sucrose phosphate synthase in dehydration induced sucrose accumulation in the resurrection plant Xerophyta humilis. *Unpublished* www.ncbi.nlm.nih.gov (2010).

70. Bennett, T. *et al.* Paralogous Radiations of PIN Proteins with Multiple Origins of Noncanonical PIN Structure. *Mol. Biol. Evol.* **31,** 2042–2060 (2014).

71. Neumann, A. *et al.* Characterisation of Galactinol synthase II from the resurrection plant Xerophyta viscosa (Baker). *Unpublished* www.ncbi.nlm.nih.gov (2016).

72. Glantz, S. T. *et al.* Functional and topological diversity of LOV domain photoreceptors. *Proc. Natl. Acad. Sci.* **113,** E1442–E1451 (2016).
